# 15-lipoxygenase blockade switches off pan-organ ischaemia-reperfusion injury by inhibiting pyroptosis

**DOI:** 10.1186/s43556-025-00325-z

**Published:** 2025-10-10

**Authors:** Jie Li, Hailong Zhang, Mengmeng Dai, Yongpan Huang

**Affiliations:** 1 Central Laboratory, The Affiliated Changsha Hospital of Xiangya School of Medicine, Central South University, Changsha, 410005 China; 2 Joint National Laboratory for Antibody Drug Engineering, School of Medicine, Henan University, Kaifeng, 475004 China; 3 Medical School, Changsha Social Work College, Changsha, 410004 China

**Keywords:** ALOX15, 15-HpETE, Thiolox, Lipid peroxidation, Inflammasome, Pyroptosis, Ischemia-reperfusion injury

## Abstract

Even with reperfusion therapy, ischemia-reperfusion (I/R) injury remains to be a major driver of organ failure associated with myocardial infarction, stroke, and liver transplantation, with effective therapeutic targets still elusive. Using in vitro hypoxia/reoxygenation (H/R) models, we discovered that the pharmacological inhibition of 15-lipoxygenase (ALOX15) by thiolox effectively mitigates myocardial I/R injury. While ALOX15—a well-established promoter of lipid peroxidation and ferroptosis—has been extensively studied in cardiac I/R, its involvement in multi-organ I/R injury and non-ferroptotic cell death has not been thoroughly investigated. To address this, we employed I/R models in three vital organs and found that either global deletion of *Alox15* or its specific loss in hematopoietic cells (*Alox15*^*ΔH*^) consistently led to a reduction in infarct volume and improvement in function across the heart, brain, and liver. Mechanistically, this protection arose from the inhibition of pyroptosis. The underlying cascade involves mitochondrial reactive oxygen species (ROS) activating ALOX15 during reperfusion, which produces 15-HpETE, leading to a collapse of mitochondrial membrane potential (ΔΨm) and subsequent IP3R-mediated calcium (Ca^2^⁺) efflux. This Ca^2^⁺ surge initiates the assembly of NLRP3 inflammasome, driving GSDMD-dependent pyroptosis. Thus, ALOX15 acts as a keystone regulator bridging oxidative stress to pyroptosis via a mitochondria-Ca^2^⁺-pyroptosis axis. This axis functions independently of the organ type and is transmitted through both parenchymal and hematopoietic cells, suggesting that thiolox and targeted ALOX15 inhibition could be viable strategies for protecting multiple organs from I/R injury.

**Supplementary Information:**

The online version contains supplementary material available at 10.1186/s43556-025-00325-z.

## Introduction

Ischemia–reperfusion (I/R) injury is the common pathological denominator underlying acute myocardial infarction, ischemic stroke and liver graft failure [1]. Although prompt restoration of blood flow is essential for treatment, this reperfusion process can unintentionally exacerbate tissue damage due to I/R injury [1]. Even with improvements in revascularization techniques and medication, lingering I/R injury still contributes to the advancement of organ failure [1].

The pathophysiology of I/R injury is multifaceted, involving a surge in reactive oxygen species (ROS), calcium overload, mitochondrial dysfunction, and sterile inflammation, which ultimately lead to various forms of regulated cell death [2, 3]. Traditionally, studies have focused on apoptosis and necrosis as the primary culprits, but recent insights have illuminated the significant contributions of necroptosis, ferroptosis, and pyroptosis in amplifying tissue injury [4, 5]. Pyroptosis, in particular, stands out as a pro-inflammatory programmed necrosis pathway, characterized by the activation of inflammasomes, the cleavage of gasdermin D (GSDMD) by Caspase-1/11, and the release of interleukin-1β (IL-1β) and IL-18, which perpetuate a vicious cycle of inflammation and cell lysis [6]. In the context of myocardial I/R, pyroptosis has been implicated in the loss of cardiomyocytes and microvascular dysfunction, exacerbating infarct size and impairing contractile function [7]. Likewise, in cerebral and hepatic I/R, it contributes to neuronal death and liver cell necrosis, connecting localized damage to systemic inflammatory responses [8]. Galluzzi et al. delineate the interplay of these death modalities, emphasizing how their convergence under oxidative stress dictates organ outcomes [9]. However, despite the potential of inhibitors of apoptosis (e.g., caspase blockers) and necroptosis (e.g., necrostatin-1) in preclinical models, their application in human cases remains limited, underscoring a need to target upstream regulators that orchestrate these pathways.

Lipoxygenases (LOXs), enzymes that catalyze the dioxygenation of polyunsaturated fatty acids to generate bioactive lipid mediators, have emerged as key players in oxidative stress and inflammation during I/R [10, 11]. Among them, arachidonate 15-lipoxygenase (ALOX15) is particularly intriguing due to its induction under hypoxic conditions and its role in producing hydroperoxyeicosatetraenoic acids (HpETEs), such as 15-HpETE, which fuel lipid peroxidation and mitochondrial damage [12]. Previous studies have primarily linked ALOX15 to ferroptosis, an iron-dependent cell death mode involving lipid radical chain reactions in myocardial I/R models [13]. The genetic deletion of *Alox15* or its pharmacological inhibition with compounds like ML351 has been shown to attenuate ferroptotic damage in cardiomyocytes, helping to maintain mitochondrial integrity and reduce ROS accumulation [13]. However, the potential role of ALOX15 in other cell death pathways, such as pyroptosis, has not been incompletely explored. Besides, the focus on specific organs has hindered a comprehensive understanding of the fundamental mechanisms behind I/R injury, with limited information available regarding *Alox15*'s involvement in cerebral and hepatic I/R contexts [14].

This research highlights the crucial function of ALOX15 in facilitating pyroptosis in various organs during I/R events. Through *in-vitro* screening, we identified thiolox, a novel ALOX15 inhibitor [15], as an effective shield against cell death triggered by hypoxia and reoxygenation. In vivo, administration of thiolox significantly inhibited myocardial I/R injury. In addition, the genetic *Alox15* deletion led to a notable decrease in infarct size and functional impairments in mouse models of myocardial, cerebral, and hepatic I/R. Mechanistically, we demonstrate that ALOX15 promotes pyroptosis via 15-HpETE-mediated mitochondrial dysfunction and Ca^2^⁺ overload, culminating in inflammasome assembly and GSDMD cleavage. This axis is conserved across organs and is intensified by hematopoietic ALOX15, where pyroptosis in macrophage leads to paracrine death in parenchymal cells. Our results reveal a common pathway linking lipid peroxidation and pyroptosis, suggesting that targeting ALOX15 could offer a comprehensive approach to improve reperfusion outcomes, potentially transforming treatment strategies for acute myocardial infarction, stroke, and organ transplantation. By filling this mechanistic void, our work paves the way for the development of targeted inhibitors like thiolox, fostering precision medicine in I/R syndromes and ultimately lessening the global toll of cardiovascular morbidity.

## Results

### Thiolox rescues myocardial I/R in vitro and in vivo by targeting ALOX15

**Fig. 1:**
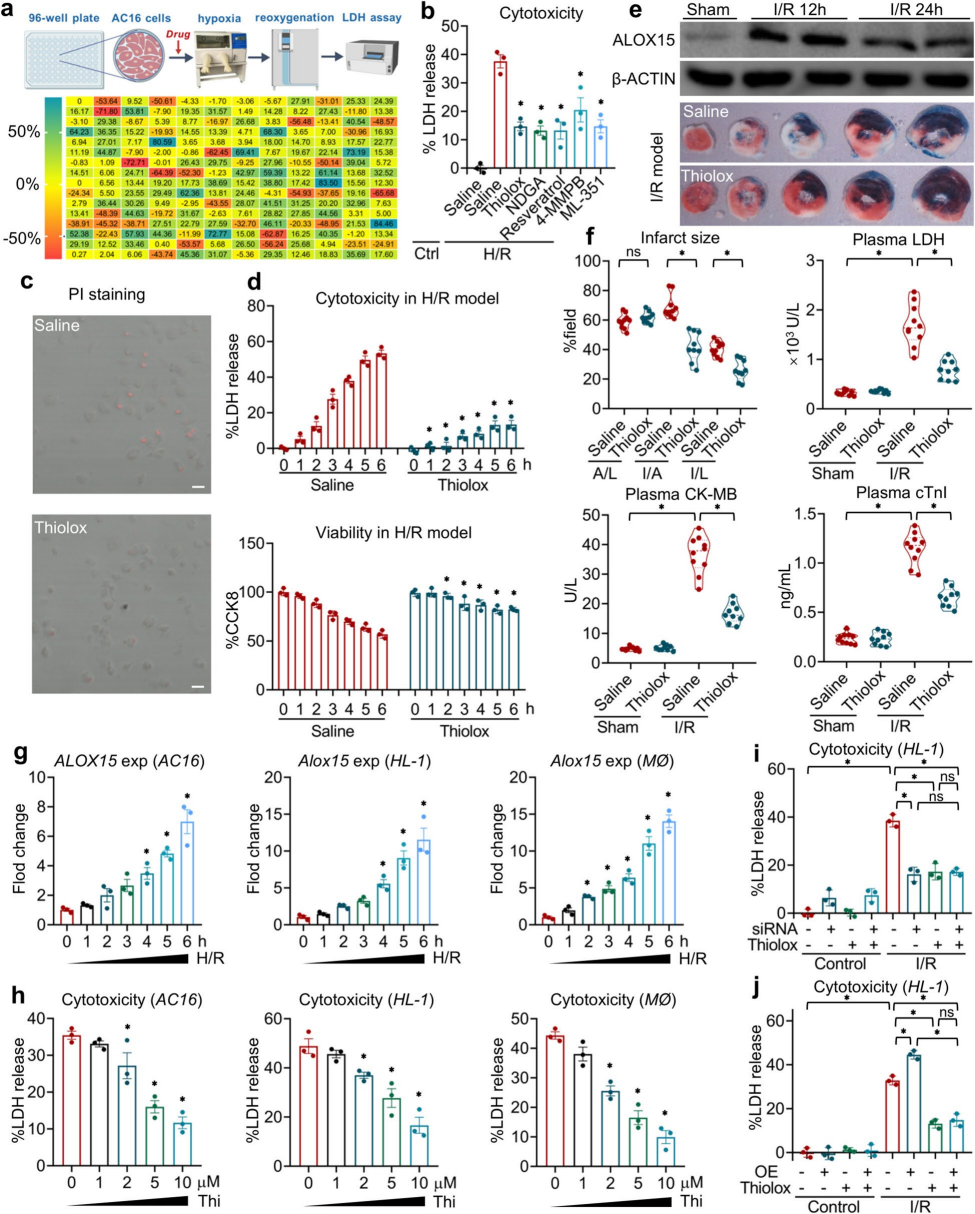
Thiolox inhibits myocardial H/R injury by inhibiting ALOX 15. **a** Heatmap of inhibition of chemicals (190 chemicals with doses of 10 μM) on H/R-induced death of *AC16* cells. **b** Inhibitory effects of thiolox (10 μM), NDGA (10 μM), resveratrol (10 μM), 4-MMPB (10 μM) and ML351 (10 μM) on H/R-induced death of AC16 cells. **c** PI staining of *AC16* cells treated with thiolox (10 μM) and challenged with H/R (Scale bar: 50 μm). **d** Cytotoxicity and viability of *AC16* cells treated with thiolox (10 μM) and challenged with 6 h of hypoxia and 1 h reoxygenation. **e**-**f** Mice were challenged with 40 min of ischemia plus 24 h of reperfusion on the left coronary artery, and thiolox (10 mg/kg) was administrated 30 min before the reperfusion (*i.v.*). **e** ALOX15 protein level and Even’s blue/TTC staining the heart. **f** Heart infarct size and plasma levels of LDH, CK and cTnI. A/L, AAR/LR. I/A, IS/AAR. I/L, IS/LV. **g** *Alox15* expression of *AC16* cells, *HL-1* cells and peritoneal macrophages with a challenge of gradient hypoxia (1–6 h) and reoxygenation (1 h). **h** Cytotoxicity of *AC16* cells, *HL-1* cells and peritoneal macrophages with a treatment of a gradient doses of thiolox (1–10 μM) and a challenge of H/R (6/1 h). **i**-**j** Cytotoxicity of *Alox15*-silencing or *Alox15*-overexpressing *HL-1* cells with a treatment of saline or thiolox (10 μM) and a challenge of H/R (6/1 h). Data are present as Mean ± SEM. One-way ANOVA with post-hoc tests were used for comparisons between groups. *, significant difference (*P* < 0.05)

To find the effective inhibitors of I/R-induced cardiac injury, we established an *in-vitro* system using a human immortalized cardiomyocyte (*AC16*) in a hypoxia/reoxygenation (H/R) model and screened the chemicals from our drug pool (Fig. 1a). We found that thiolox, along with other lipoxygenase inhibitors, such as nordihydroguaiaretic acid (NDGA), resveratrol, 4-MMPB and ML351, significantly retarded H/R-induced cell death (Fig. 1a and b). Besides of well-studied NDGA, 4-MMPB and ML351, Thiolox is reported to be a novel and promising candidate for treating a series of inflammatory diseases [15]. Given that its effects on myocardial infarction (MI) remain unknown, we further validated its inhibition of H/R-induced cell injury. Using propidium iodide (PI) staining to detect the membrane rupture associated cell death, we noted a marked rise in cardiomyocyte death due to H/R, which was significantly counteracted by thiolox treatment (Fig. 1c). As the duration of hypoxia increased, cardiomyocyte injury progressively intensified, as evidenced by elevated lactate dehydrogenase (LDH) release and decreased cell viability (Fig. 1d). These detrimental effects were considerably alleviated with thiolox administration (Fig. 1d), suggesting that this compound effectively inhibits cell death associated with H/R.

As ALOX15 expression is boosted after a cardiac I/R challenge, we administered thiolox to mice subjected to I/R to assess its *in-vivo* inhibitory properties (Fig. 1e). The results indicated that both the saline- and thiolox-treated mice exhibited comparable ratios of area at risk (AAR) to left ventricle (LV, AAR/LV, A/L), meaning no bias in the I/R challenge between the two groups (Fig. 1f). Administration of thiolox robustly reduced myocardial damage caused by I/R. The infarct size (IS/AAR, I/A; and IS/LV, I/L) in the hearts of thiolox-treated mice was significantly smaller than that of those receiving saline (Fig. 1f). As myocardial injury releases LDH into the bloodstream and boosts the circulating levels of CK and cTnI, we measured the plasma levels of LDH, CK and cTnI in the different groups of mice. Correspondingly, the I/R-induced elevations in LDH, CK and cTnI, reflecting the extent of myocardial injury, were significantly attenuated by administrating thiolox (Fig. 1f), thereby confirming the protective effects of this compound on MI. Thus, these findings suggest that thiolox is an effective inhibitor of myocardial I/R.

Since thiolox acts as a specific inhibitor of ALOX15 [15], we compared the expression levels of *ALOX15* in different cell types after H/R challenge. We found that in an *in-vitro* model, H/R exposure led to a time-dependent increase in *ALOX15* expression levels in *AC16* cells: the prolonged hypoxic conditions resulted in heightened *ALOX15* expression (Fig. 1g). This pattern was also observed in *HL-1* cells derived from mice and in macrophages (Fig. 1g), indicating that H/R upregulates the expression of *ALOX15* across various cell types. Moreover, thiolox treatment significantly reduced H/R-induced cell death in a dose-dependent manner across these three types of cells (Fig. 1h). To further confirm if the observed inhibitory effects were due to ALOX15 blockade, we employed siRNA to knock down *Alox15* or used a plasmid to overexpress *Alox15* in *HL-1* cells. In the H/R model, silencing *Alox15* led to a notable decrease in cell death, while overexpression of *Alox15* resulted in a significant increase in cell death compared to control groups (Fig. 1i and j). Notably, thiolox exhibited inhibitory effects similar to *Alox15* knockdown on H/R-induced cell death, with no additional impact observed regardless of whether *Alox15* was silenced or overexpressed (Fig. 1i and j). Taken together, these results demonstrate that thiolox alleviates H/R-induced cell death by specifically targeting ALOX15.

### Genetical deletion of *Alox15* improves I/R-induced injury and dysfunction

**Fig. 2:**
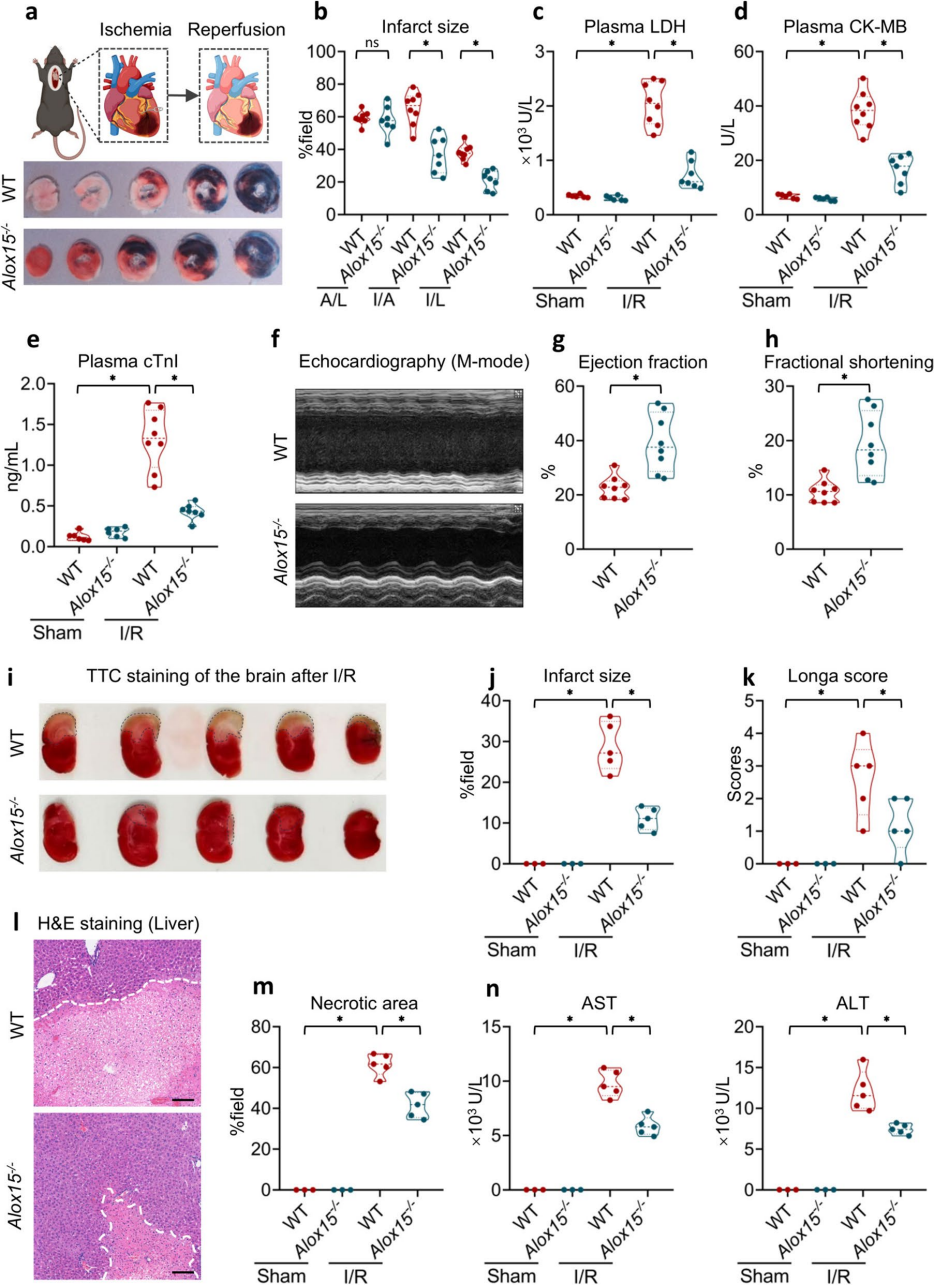
*Alox15* deficiency protects against I/R-induced injury across pan-organs. **a-h** WT and *Alox15*^−/−^ mice were challenged with 40 min of ischemia and 24 h of reperfusion on the left coronary artery. **a** Even’s blue/TTC staining of the heart 24 h after reperfusion. **b** Infarct size of the heart. Plasma levels of LDH (**c**), CK (**d**) and cTnI (**e**). **f**-**h** Echocardiographic examination of the left ventricle of heart challenged with I/R for 4 weeks. M-mode echocardiography (**f**), EF (**g**) and FS (**h**) of the left ventricle. **i-k** WT and *Alox15*.^−/−^ mice were subjected to t-MCAO for 60 min and reperfusion for 24 h. TTC staining (**i**, Scale bar: 200 μm) and infarct size of the brain (**j**). Longa score indicating neurological function (**k**). **l-n** Hepatic I/R induced 1 h ischemia and 6 h reperfusion of the liver. H&E staining of the liver (**l**), Necrotic area (**m**) and plasma level of AST and ALT (**n**). Data are present as Mean ± SEM. One-way ANOVA with post-hoc tests were used for comparisons between groups. *, significant difference (*P* < 0.05)

To validate the role of ALOX15 in myocardial I/R, we genetically knocked out *Alox15* in mice and subjected both WT and *Alox15*-deficient mice to the I/R model (Fig. 2a). It was found that both the infarct size was notably reduced in *Alox15*^−/−^ mice compared to WT mice (Fig. 2a and b), without discernible bias observed in the I/R model in either group (Similar A/L), indicating a protective effect associated with *Alox15* depletion. Furthermore, heart injury-related LDH release was significantly lower upon the deletion of *Alox15* (Fig. 2c). Blood levels of CK and cTnI also showed a marked decrease in *Alox15*^−/−^ mice (Fig. 2d and e). Additionally, MI typically induces chronic remodeling of the heart. To determine the effects of ALOX15 on these chronic cardiac alterations, we monitored WT and *Alox15*^−/−^ mice subjected to I/R for 4 weeks and assessed their cardiac function. We found that the absence of *Alox15* substantially mitigated I/R-induced chronic injury (Fig. 2f-g). Echocardiographic assessments revealed that *Alox15* deficiency preserved the left ventricle’s pumping function (Fig. 2f). Moreover, the ejection fraction (EF) and fractional shortening (FS), indicators of left ventricle function, were significantly elevated in *Alox15*-deleting mice (Fig. 2g and h). Collectively, these results imply that blocking ALOX15 could serve as a promising therapeutic strategy to protect against cardiac injury resulting from MI.

Beyond the heart, cerebral I/R injury is a major clinical burden in cardiovascular medicine [16], and I/R damage to grafts remains a principal cause of primary non-function after liver transplantation [17, 18]. To test whether ALOX15 acts as a convergent driver of I/R injury across organs, we subjected WT and *Alox15*-knockout mice to cerebral and hepatic I/R paradigms. In a transient middle-cerebral-artery occlusion model, *Alox15*^−/−^ mice exhibited significant reduction in infarct volume compared to their WT littermates (Fig. 2i and j). This structural protection translated into substantial functional recovery, as evidenced by lower Longa neurological scores (Fig. 2k), echoing the cardioprotective phenotype we previously described. Likewise, in a warm hepatic I/R scenario, *Alox15* deficiency curtained necrotic areas (Fig. 2l and m) and blunted hepatocellular injury, as shown by decreased levels of plasma aspartate aminotransferase (AST) and alanine aminotransferase (ALT) (Fig. 2n). Overall, these findings across multiple organs highlight ALOX15 as a common upstream instigator in I/R injury and demonstrate that its genetic suppression confers robust, organ-wide protection against the devastating consequences of reperfusion.

### Genetic deletion of *Alox15* inhibits pyroptosis during H/R

**Fig. 3:**
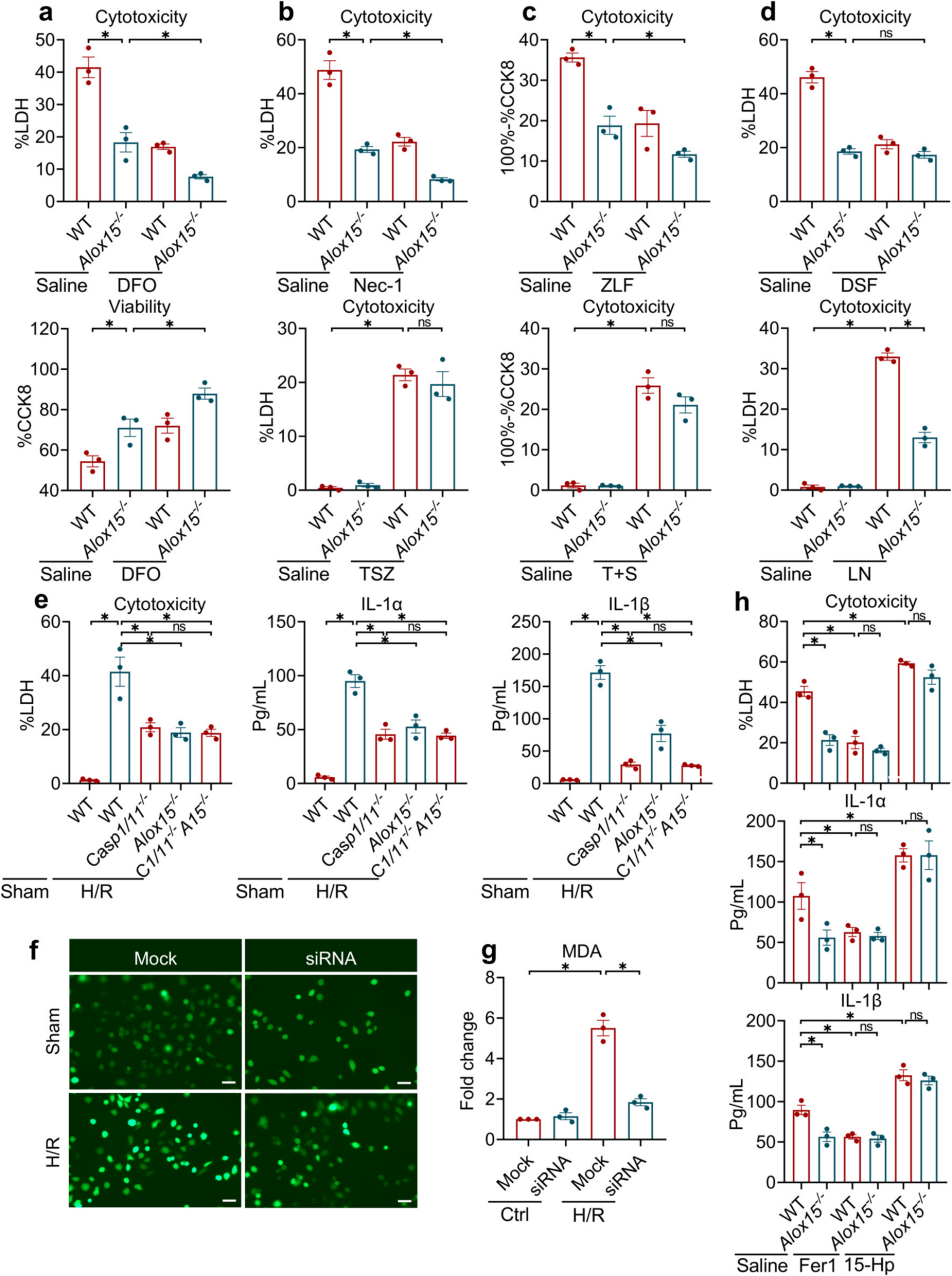
ALOX15 blockade inhibits I/R injury by inhibiting pyroptosis. **a-d** Cytotoxicity and viability of WT and *Alox15*^−/−^ macrophages: (**a**) treated with DFO (iron chelator) or H/R; (**b**) treated with Nec-1 (necroptosis inhibitor) under a challenge of H/R or TSZ (necroptosis inducer); (**c**) treated with ZLF (apoptosis inhibitor) under a challenge of H/R or T + Z; (**d**) treated with DSF (disulfiram, pyroptosis inhibitor) and LPS + Nigerincin (pyroptosis inducer) under a challenge of H/R and pytoptosis. **e** Cytotoxicity and IL-1α/β release of *Casp1/11*^*−/−*^, *Alox15*^−/−^ and *Casp1/11*^*−/−*^*Alox15*^−/−^ macrophages challenged with H/R. **f**-**g** ROS staining (**f**, Scale bar: 50 μm) and MDA level (**g**) of *AC16* cells treated with *Alox15* siRNA and challenged with H/R (6/1 h). **h** Cytotoxicity and IL-1α/β release of WT and *Alox15*^−/−^ macrophages treated with Ferrostain-1 (Fer1) and 15-HpETE under a challenge of H/R. *C1/11*^*−/−*^*A15*^−/−^, *Casp1/11*^*−/−*^*Alox15*^−/−^. Data are present as Mean ± SEM. One-way ANOVA with post-hoc tests were used for comparisons between groups. *, significant difference (*P* < 0.05)

I/R injury is executed through an intricate interplay of various programmed cell-death modalities, including ferroptosis, pyroptosis, necroptosis, and apoptosis [19]. Among these, ferroptosis is uniquely driven by iron-catalyzed Fenton chemistry and subsequent lipid-peroxidative chain reactions [20, 21]. Given that ALOX15 is a canonical enzymatic amplifier of this process, we first interrogated its role in H/R using *Alox15*^*−/−*^ cells, with or without the iron chelator deferiprone (Fig. 3a). As expected, iron chelation reduced cell death caused by H/R, but the absence of *Alox15* led to an even greater decrease. This synergistic suppression hinted that ALOX15 may orchestrate death pathways beyond just ferroptosis. To dissect these alternate pathways, we subjected *Alox15*^*−/−*^ cells to a panel of selective death inhibitors during H/R, as well as with specific triggers for necroptosis, apoptosis, and pyroptosis (Fig. 3b-d). Either blocking necroptosis with Nec-1 or inhibiting apoptosis with Z-LEHD-FMK conferred synthetically inhibitory effects on H/R lethality in the context of *Alox15* deficiency (Fig. 3b and c). However, *Alox15* knockout showed only a slight impact on stimulator-induced pure necroptotic and apoptotic death (Fig. 3b and c), indicating the marginal effects of ALOX15 in these processes. In stark contrast, deleting *Alox15* failed to further enhance survival in disulfiram-treated (a pyroptosis inhibitor) cells under H/R (Fig. 3d). Moreover, *Alox15* deletion robustly suppressed canonical NLRP3-mediated pyroptosis, highlighting its crucial role in this form of cell death. To corroborate pyroptosis as the dominant target in H/R, we generated double-knockout (*Alox15*^*−/−*^*Casp1/11*^*−/−*^) cells. While single deletion of either *Casp1/11* or *Alox15* resulted in similar reductions in cell death during H/R, the double knockout did not provide any additional benefit (Fig. 3e). Concomitant release of IL-1α/β, which are key cytokines associated with pyroptosis, mirrored these findings (Fig. 3e). Collectively, these data indicate pyroptosis as the primary cell death influenced by *Alox15* during H/R.

ALOX15 functions as an enzyme that promotes lipid peroxidation. To assess this, we measured levels of ROS and malondialdehyde (MDA) as lipid-oxidation surrogates. H/R provoked a marked increase in ROS and MDA, which were eliminated by silencing *ALOX15* (Fig. 3f and g). The lipid-radical scavenger ferrostatin-1 phenocopied the effects of *Alox15* deletion, suppressing cell death caused by H/R and pyroptosis, as well as IL-1α/β release (Fig. 3h). Conversely, supplementation with the ALOX15 metabolite 15-HpETE amplified pyroptotic cell death and cytokine secretion (Fig. 3h). In sum, ALOX15 oxidizes lipids to ignite pyroptotic cell death during H/R stress; genetic or pharmacologic interruption of this lipid-oxidation axis may preserve cellular viability by preventing inflammatory cell death.

### Deleting *Alox15* protects against I/R in the heart, brain and liver by inhibiting pyroptosis

**Fig. 4:**
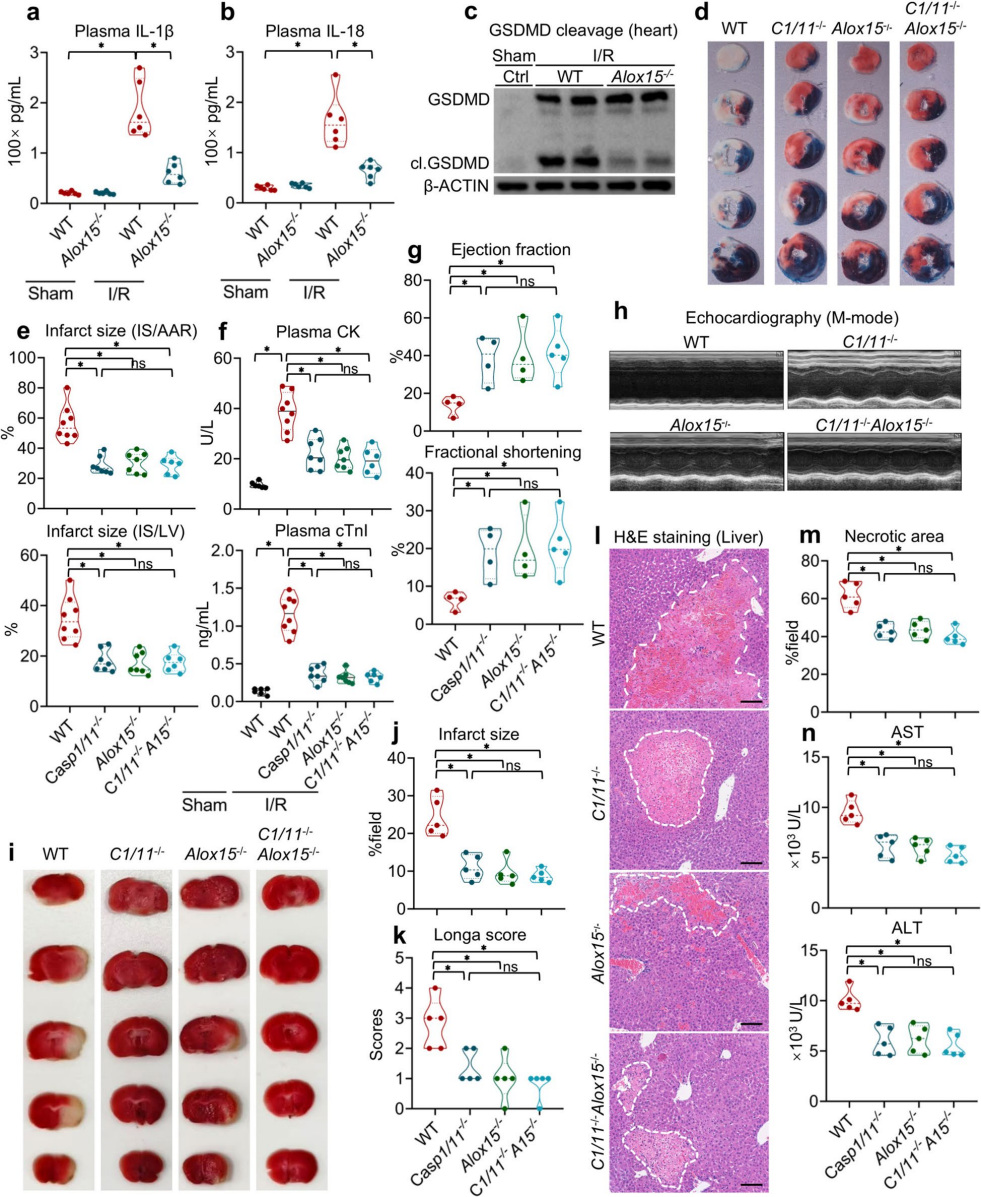
Deleting *Alox15* protects against I/R-induced injury across pan-organs by inhibiting pyroptosis. **a-c** Plasma level of IL-1β (**a**) and IL-18 (**b**), and GSDMD cleavage (**c**) of the heart from control and I/R-challenged WT and *Alox15*^−/−^ mice. **d-f** WT, *Casp1/11*^*−/−*^, *Alox15*^−/−^ and *Casp1/11*^*−/−*^*Alox15*^−/−^ mice were challenged with 40 min of ischemia and 24 h of reperfusion on the left coronary artery. Even’s blue/TTC staining of the heart 24 h after reperfusion (**d**). Infarct size of the heart (**e**). Plasma levels of CK and cTnI (**f**). **g-h** Echocardiographic examination of the left ventricle of heart challenged with I/R for 4 weeks. EF and FS (**g**) of the left ventricle, M-mode echocardiography (**h**). **i-k** WT and *Alox15*.^−/−^ mice were subjected to t-MCAO for 60 min and reperfusion for 24 h. TTC staining (**i**) and infarct size of the brain (**j**). Longa score indicating neurological function (**k**). **l-n** Hepatic I/R induced 1 h ischemia and 6 h reperfusion of the liver. (**l**) H&E staining of the liver (Scale bar: 200 μm), Necrotic area (**m**) and plasma level of AST and ALT (**n**). Data are present as Mean ± SEM. One-way ANOVA with post-hoc tests were used for comparisons between groups. *, significant difference (*P* < 0.05)

To further investigate the role of ALOX15 and pyroptosis in vivo, we assessed critical markers of pyroptosis, including the secretion of IL-1β and IL-18, along with GSDMD cleavage, in the heart under both I/R stress and normal conditions. The results demonstrated that the absence of *Alox15* significantly reduced the plasma levels of IL-1β and IL-18 (Fig. 4a and b) and diminished GSDMD cleavage in the heart (Fig. 4c), indicating that ALOX15 facilitates pyroptosis in MI. To determine the role of pyroptosis in ALOX15-associated MI, we generated knockout mice for *Alox15* and/or *Casp1/11*, and subjected WT, *Alox15*^*−/−*^*, Casp1/11*^*−/−*^ and *Casp1/11*^*−/−*^*Alox15*^*−/−*^ mice to I/R challenges. We found that *Casp1/11* knockout showed similar inhibitory effects on I/R-induced infarct size as those seen with *Alox15* deficiency (Fig. 4d and e). Moreover, the dual knockout of *Casp1/11* and *Alox15* did not confer additional protection against MI (Fig. 4d and e). Similar trends were observed in the levels of CK and cTnI (Fig. 4f). Additionally, echocardiography revealed that indices of EF and FS (Fig. 4g), as well as the left ventricle pulsation capability (Fig. 4h), were higher in *Alox15*^*−/−*^ and *Casp1/11*^*−/−*^ mice, with no further improvement in *Casp1/11*^*−/−*^*Alox15*^*−/−*^ mice. Taken together, these findings suggest that ALOX15 promotes MI by enhancing pyroptosis.

To investigate the conservation of the ALOX15-pyroptosis pathway beyond cardiac tissue, we examined WT, *Alox15*^*−/−*^*, Casp1/11*^*−/−*^ and *Casp1/11*^*−/−*^*Alox15*^*−/−*^ mice using cerebral and hepatic I/R models. In the brain, both *Alox15* and *Casp1/11* single deletions reduced infarct size after a temporary occlusion of the middle cerebral artery, with no additional benefit observed in the dual knockouts (Fig. 4i and j). The functional recovery paralleled the structural preservation: Longa neurological scores significantly fell in *Alox15*^*−/−*^ and *Casp1/11*^*−/−*^ mice, respectively, and remained no further reduction in the dual-deficient cohort (Fig. 4k). Besides, the liver exhibited comparable results. Following I/R, necrotic areas similarly shrank in both *Alox15*^*−/−*^ and *Casp1/11*^*−/−*^ livers (Fig. 4l and m), and the levels of ALT and AST mirrored these histological changes, each dropping by approximately 50% in the single knockout. No further decrement was found in the dual knockouts (Fig. 4n). Thus, across three vital organs—heart, brain, and liver—genetic ablation of either *Alox15* or *Casp1/11* provides equivalent, non-redundant protection, while their combined deletion yields no additive benefit. These consistent results highlight the ALOX15-pyroptosis axis as a fundamental, organ-independent mechanism that contributes to the overall impact of I/R injury.

### Deleting hematopoietic ALOX15 protects against I/R by reducing paracrine cell death

**Fig. 5:**
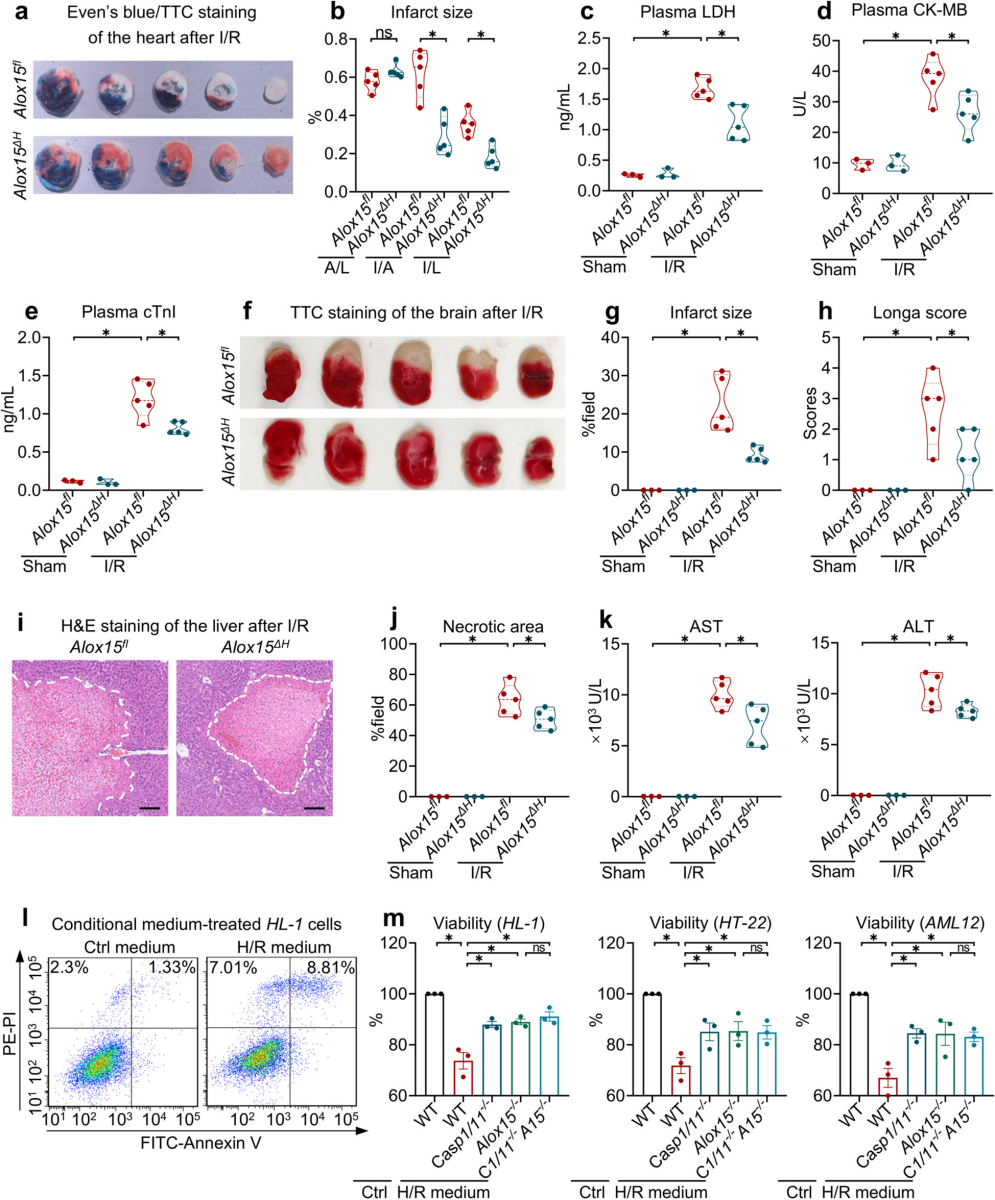
Hematopoietic deleting *Alox15* reduces paracrine cell death in I/R model. **a-e** *Alox*^*fl*^ and *Alox15*^*ΔH*^ mice were challenged with 40 min of ischemia and 24 h of reperfusion on the left coronary artery. **a** Even’s blue/TTC staining of the heart 24 h after reperfusion. **b** Infarct size of the heart. Plasma levels of LDH (**c**), CK (**d**) and cTnI (**e**). **f**-**h** *Alox*^*fl*^ and *Alox15*^*ΔH*^ mice were subjected to t-MCAO for 60 min and reperfusion for 24 h. TTC staining (**f**) and infarct size of the brain (**g**). Longa score indicating neurological function (**h**). **i-k** Hepatic I/R induced 1 h ischemia and 6 h reperfusion of the liver. (**i**) H&E staining of the liver (Scale bar: 200 μm). Necrotic area (**j**) and plasma level of AST and ALT (**k**). **l-m** WT, *Casp1/11*^*−/−*^, *Alox15*^−/−^ and *Casp1/11*^*−/−*^*Alox15*.^−/−^ macrophages were challenged with H/R (6/1 h), and the conditioned medium was applied to *HL-1*, *HT-22* and *AML-12* cells for 12 h. **l** Cytometry indicating conditioned medium-induced cell death. **m** Cytotoxicity of *HL-1, HT-22* and *AML-12* cells under a treatment of conditional medium. Data are present as Mean ± SEM. One-way ANOVA with post-hoc tests were used for comparisons between groups. *, significant difference (*P* < 0.05)

Our preceding data revealed that macrophages undergo pyroptosis during H/R and that *Alox15* deletion blunts this response (Fig. 3). Increasing evidence suggests that immune cells, especially macrophages, act as central amplifiers of I/R pathology [22–24]. Once macrophages undergo pyroptosis, they can induce cell death in adjacent parenchymal cells through a mechanism known as paracrine pyroptosis [25]. This led us to investigate whether ALOX15 in the hematopoietic system serves as a crucial trigger. To explore this, we generated *Alox15*^*ΔH*^ mice by crossing *Alox15*-flox mice with *Vav*-Cre transgenics, resulting in specific *Alox15* deletion across all hematopoietic lineages. These mice were then subjected to cardiac, cerebral and hepatic I/R protocols. In the heart, under similar area-at-risk (AAR/LV), *Alox15*^*ΔH*^ mice displayed significant reduction in infarct size (IS/AAR and IS/LV) versus littermate controls (Fig. 5a and b). This reduction in infarct size corresponded with decreased plasma LDH levels (Fig. 5c), along with lower CK and cTnI release in *Alox15*^*ΔH*^ mice (Fig. 5d and e). Remarkably similar protection was noted in the brain, where *Alox15*^*ΔH*^ mice had a significant reduction in cerebral infarct volume following transient middle-cerebral-artery occlusion (Fig. 5f and g) and demonstrated improved neurological performance (Fig. 5h). In the liver, 60 min of warm ischemia followed by 6 h of reperfusion produced extensive necrosis and a sharp rise in transaminases. Hematopoietic *Alox15* deletion curtailed necrotic area and simultaneously blunted AST and ALT surges (Fig. 5i-k). These *in-vivo* observations suggest that ALOX15 signaling from hematopoietic cells plays a significant role in I/R injury. To formalize the interaction of ALOX15 with pyroptosis, we exposed WT, *Alox15*^*−/−*^*, Casp1/11*^*−/−*^, and *Casp1/11*^*−/−*^*Alox15*^*−/−*^ macrophages to H/R conditions. The conditioned medium from WT macrophages reduced the viability of *HL-1* (cardiac), *HT-22* (neuronal), and *AML-12* (hepatic) cells (Fig. 5l and m). In contrast, the medium from *Alox15*^*−/−*^ or *Casp1/11*^*−/−*^ macrophages exhibited reduced cytotoxicity, while combined deletion offered no additional advantage (Fig. 5m). Collectively, these data identify hematopoietic ALOX15 as a key regulator of macrophage pyroptosis. Once unleashed, this pathway releases cytotoxic vesicles that drive paracrine death in parenchymal cells [25], thereby exacerbating damage in the heart, brain and liver. Consequently, targeting ALOX15 in the hematopoietic compartment could be a powerful, pan-organ strategy to blunt I/R-induced organ failure.

### ALOX15 licenses inflammasome assembly to ignite pyroptosis during hypoxia/reoxygenation

**Fig. 6:**
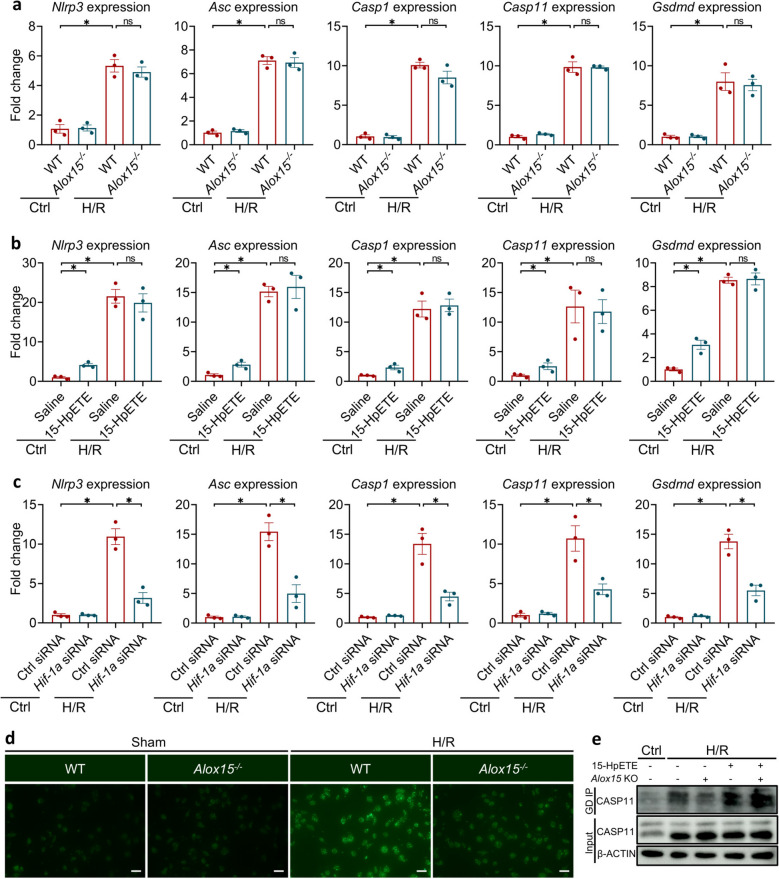
ALOX15 facilitates inflammasome assembly in H/R. **a** The expression levels of *Nlrp3*, *Asc*, *Caspase-1*, *Caspase-11* and *Gsdmd* in WT and *Alox15*^−/−^ macrophages under a challenge of H/R. **b** The expression levels of *Nlrp3*, *Asc*, *Caspase-1*, *Caspase-11* and *Gsdmd* in macrophages under a treatment of 15-HpETE and a challenge of H/R. **c** The expression levels of *Nlrp3*, *Asc*, *Caspase-1*, *Caspase-11* and *Gsdmd* in *Hif-1α*-silencing macrophages under a challenge of H/R. **d** ASC staining of WT and *Alox15*^−/−^ macrophages challenged with H/R or not (Scale bar: 50 μm). **e** Co-IP indicating the binding of GSDMD with Caspase-11 WT and *Alox15*.^−/−^ macrophages under a treatment of 15-HpETE and a challenge of H/R. Data are present as Mean ± SEM. One-way ANOVA with post-hoc tests were used for comparisons between groups. *, significant difference (*P* < 0.05)

Pyroptosis is activated when inflammasomes switch from a quiescent scaffold to an active proteolytic platform [26]. The canonical complexes comprise NLRP3, ASC and Caspase-1, while the non-canonical complexes rely on Caspase-11 sensing cytosolic lipopolysaccharide (LPS) [27–29]. The assembly proceeds in two discrete stages: (1) a transcriptional priming phase that up-regulates the levels of sensor and effector proteins, and (2) a stimulus-dependent oligomerization phase that triggers the formation ASC specks and unleashes Caspase-1/11 activation [30]. To pinpoint where ALOX15 intervenes, we first quantified key inflammasome constituents in H/R-challenged macrophages. The genetic ablation of *Alox15* left the expression levels of *Nlrp3*, *Asc*, *Caspase-1*, *Caspase-11* and the pore-forming substrate *Gsdmd* unchanged (Fig. 6a). Likewise, exogenous 15-HpETE, the principal ALOX15-derived lipid hydroperoxide, elicited only a modest increase of these components, which was significantly lower than the increase caused by H/R alone, and failed to augment expression further when superimposed on H/R (Fig. 6b). Thus, these findings exclude ALOX15 from the priming phase. Hypoxia-induced transcriptional reprogramming is primarily regulated by hypoxia-inducible factor-1α (HIF-1α) [31]. As expected, our results discovered that silencing *Hif-1α* with siRNA blunted the H/R-induced rise in inflammasome components (Fig. 6c), confirming that the priming process is HIF-1α-driven and ALOX15-independent.

We next interrogated the second, assembly step. The formation of ASC oligomers into fluorescent specks is the morphological signature of inflammasome activation. High-resolution microscopy revealed a dense field of ASC specks in WT macrophages after H/R, whereas *Alox15*^*−/−*^ cells displayed a notable reduction in speck formation (Fig. 6d). In the case of non-canonical inflammasome complexes, *Alox15* deficiency similarly decreased H/R-induced Caspase-11/GSDMD interaction, while the introduction of 15-HpETE facilitated Caspase-11/GSDMD binding (Fig. 6e). Taken together, ALOX15 does not tune the transcriptional priming of inflammasomes; rather, it selectively enhances the second signal—lipid peroxidation amplification—that converts primed sensors into fully active, speck-forming complexes, thereby precipitating pyroptotic cell death during H/R.

### ALOX15-derived 15-HpETE ignites H/R-induced pyroptosis via a mitochondria-Ca^2^⁺ signaling relay

**Fig. 7:**
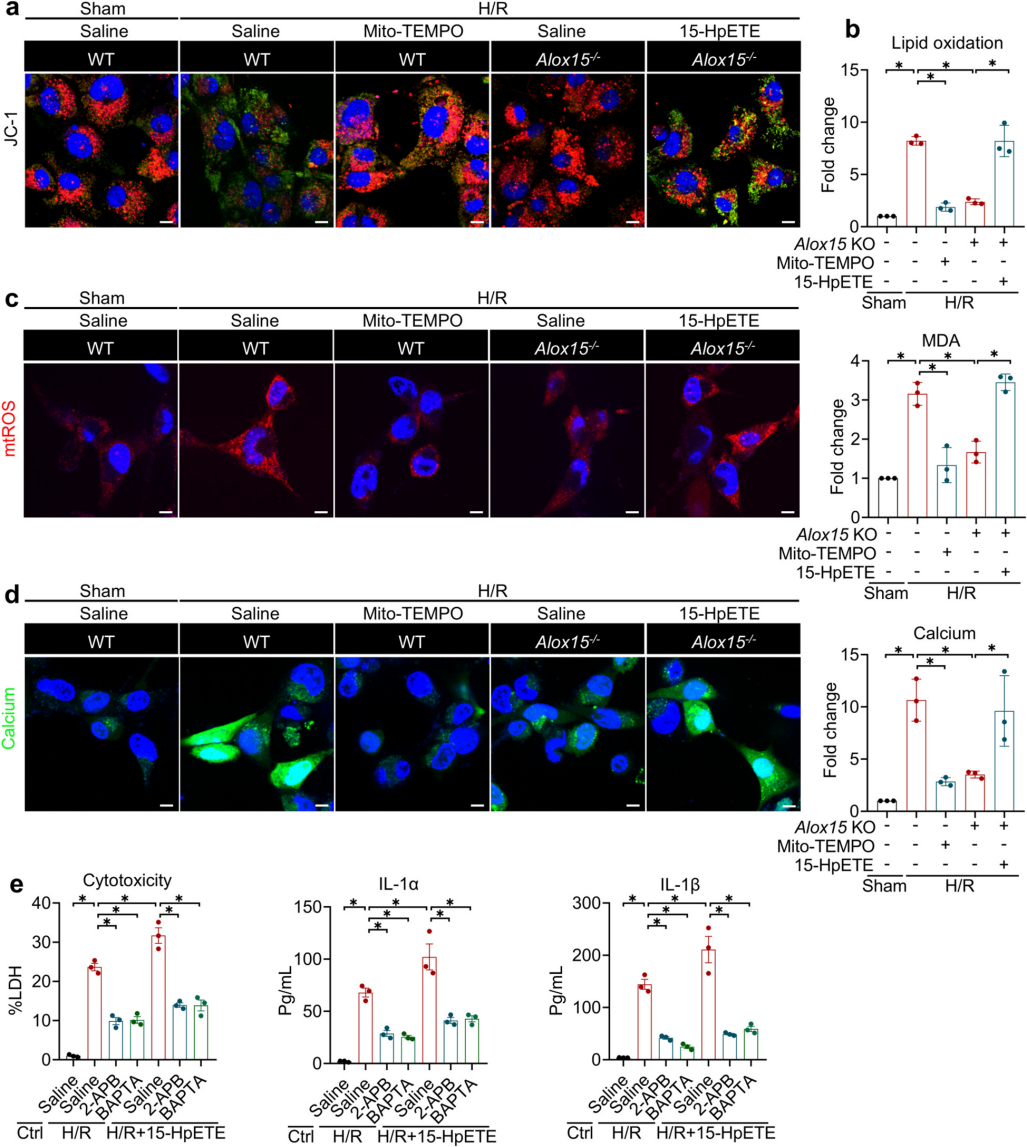
ALOX15-derived 15-HpETE facilitates pyroptosis in H/R via a mitochondria-Ca^2^⁺ Signaling. **a-d** WT and *Alox15*^−/−^ macrophages were treated with Mito-TEMPO or 15-HpETE under a challenge of H/R (Scale bar: 10 μm). **a** JC-1 staining indicating the mitochondrial function. **b** Lipid oxidation and MDA levels. **c** Mitochondrial ROS (Red). **d** Cytosolic level of calcium (Green) and quantitative analysis. **e** Cytotoxicity and IL-1α/β release of macrophages treated with 2-APB (IP3R inhibitor) and BAPTA (Ca.^2+^ chelator) and challenged with H/R. Data are present as Mean ± SEM. One-way ANOVA with post-hoc tests were used for comparisons between groups. *, significant difference (*P* < 0.05)

Mitochondria rapidly rewire the electron-transport chain during hypoxia, yet abrupt re-oxygenation leads to an electron overflow that generates mitochondrial reactive oxygen species (mtROS) and collapses membrane potential (ΔΨm), initiating downstream death cascades [32]. In line with this paradigm, H/R provoked a sharp ΔΨm drop alongside folds of surge in mtROS (Fig. 7a and b). Concomitantly, lipid peroxidation products accumulated; either the mitochondria-targeted antioxidant Mito-TEMPO or *Alox15* deletion suppressed this oxidative damage (Fig. 7c), indicating that mitochondrial dysfunction enhances ALOX15 activity. Reciprocally, the ALOX15 derivative 15-HpETE acts as a potent mitochondrial toxin [13]. Reintroducing 15-HpETE reversed *Alox15*^−/−^-restored ΔΨm loss and mtROS generation to levels similar to WT (Fig. 7a and b), confirming that 15-HpETE exacerbates mitochondrial damage in a feed-forward loop.

Mitochondrial dysfunction engages the inositol-1,4,5-trisphosphate receptor (IP3R) to trigger Ca^2^⁺ efflux from the endoplasmic reticulum, which is a prerequisite for Ca^2^⁺-dependent NLRP3 inflammasome assembly and pyroptosis [33]. Indeed, H/R evoked a dramatic cytosolic Ca^2^⁺ overload that was largely abolished in *Alox15*^*−/−*^ macrophages, and 15-HpETE re-supply reversed this protection, restoring Ca^2^⁺ to those observed in WT cells (Fig. 7d). To causally link IP3R-mediated Ca^2^⁺ release to pyroptosis induced by 15-HpETE, we treated cells with the IP3R antagonist 2-APB or the membrane-permeable Ca^2^⁺ chelator BAPTA-AM. Both interventions potently blocked H/R-induced and 15-HpETE-facilitated pyroptotic cell death (Fig. 7e). Collectively, our findings delineate a self-amplifying axis where H/R-damaged mitochondria activate ALOX15, leading to the production of 15-HpETE that inflicts further mitochondrial injury. This cascade results in an overload of cytosolic Ca^2^⁺ mediated by IP3R, which nucleates the NLRP3 inflammasome and initiates pyroptosis.

## Discussion

The present study integrates pharmacological, genetic, and cell-specific evidence to reposition ALOX15 as a master lipid-peroxidative switch that drives pyroptosis, rather than ferroptosis alone, across cardiac, cerebral, and hepatic I/R injury. Three conceptual advances emerge: (i) the discovery of a mitochondria-15-HpETE-Ca^2^⁺ pathway that licenses inflammasome assembly without altering transcriptional priming; (ii) evidence that the ALOX15/pyroptosis axis is applicable across different organs; and (iii) proof-of-principle that hematopoietic ALOX15 fuels a paracrine cell death circuit linking macrophages to parenchymal cells. These findings refine and extend prior work that primarily associated ALOX15 with ferroptotic cardiomyocyte death [13], providing a unifying framework for multi-organ protection.

Earlier landmark reports attributed I/R injury to cardiomyocyte death through ALOX15-mediated ferroptosis [13]. By dissecting cell death modalities, we uncovered ALOX15’s orchestration of pyroptosis during H/R. I/R injury involves a symphony of programmed deaths—ferroptosis, pyroptosis, necroptosis, and apoptosis—each with distinct triggers and effectors [34]. Although ALOX15 canonically amplifies ferroptosis via iron-dependent lipid peroxidation, our experiments using iron chelators like deferiprone revealed additive protection with *Alox15* deletion, hinting at a more extensive role. Selective inhibitors pinpointed pyroptosis as the dominant pathway involved: the effects of *Alox15* knockout mirrored of those observed with disulfiram (a pyroptosis inhibitor), which suppressed NLRP3-mediated pyroptosis and abrogated IL-1α/β release, with no further benefit in *Casp1/11* &*Alox15* double knockouts. Beyond ferroptosis, pyroptosis plays a pivotal role in I/R injury pathogenesis [5]. Previous clinical studies have indicated an upregulation of pyroptosis alongside elevated levels of cytokines IL-1β and IL-18 in patients with MI [35, 36]. Although the NLRP3 inflammasome primarily activates and induces pyroptosis in non-cardiomyocytes, it exacerbates both acute and chronic cardiac injury during I/R [36]. Animal studies have shown that blocking the canonical inflammasome by deleting NLRP3 or ASC reduces infarct size and preserves cardiac function in mice undergoing I/R [37, 38]. Notably, a recent study demonstrates that Caspase-11, a component of the non-canonical inflammasome and an intracellular LPS sensor, that is logistically unrelated to sterile ischemic diseases, is highly involved in the pathogenesis of MI and induces cell death in both cardiomyocytes and non-cardiomyocytes [39]. Blocking Caspase-11 markedly reduces I/R-induced infarct size and cardiac remodeling [39]. Consistent with this, our study shows that H/R upregulates inflammasome components and pyroptosis-associated cytokines, while the depletion of both *Caspase-1* and *Caspase-11* effectively inhibits cytokine release enhanced by H/R and mitigates cardiac injury from I/R, emphasizing the critical role of pyroptosis in ischemic cardiac diseases. We further confirmed that ALOX15-derived oxidized lipids serve as an essential secondary signal, amplifying mitochondrial ROS and precipitating IP3R-mediated Ca^2^⁺ efflux from the endoplasmic reticulum, which is the obligate step for NLRP3 oligomerization. This model implies that lipid peroxides act as damage-associated molecular patterns (DAMPs), facilitating inflammasome oligomerization [40, 41]. Consequently, ALOX15 emerges as a convergence point where oxidative stress transitions to inflammatory signaling, representing a paradigm shift in I/R pathology.

The ALOX15/pyroptosis regulatory axis is a conserved, organ-spanning mechanism underlying I/R injury. In the heart, our data corroborate Ma et al. [42], who linked ALOX15-mediated 15-HpETE-PE accumulation to ferroptotic death of cardiomyocytes. We further extend their model by showing that these oxidized lipids also facilitate NLRP3 inflammasome assembly and subsequent pyroptosis. In the brain, while Gaberel et al*.* associated ALOX15 with the disruption of the oxidative blood-brain barrier [43], our research demonstrates that ALOX15 suppression affords both structural and functional neuroprotection by preventing pyroptosis. In the liver, where I/R injury remains a major barrier to successful transplantation, Jia et al. reported that pharmacological ALOX15 blockade attenuates graft dysfunction [44]. Our results integrate this finding with evidence that ALOX15 propagates hepatic pyroptosis. Collectively, ALOX15 acts as a conserved enhancer of pyroptosis across the heart, brain, and liver, highlighting opportunities for tissue-tailored therapeutic targeting.

Moreover, hematopoietic *Alox15* blockade also protects against I/R injury across these organs. Immune cells are active executors of I/R injury rather than passive bystanders [1]. Within minutes of reperfusion, tissue-resident macrophages and neutrophils that are sequestered in the bloodstream sense I/R-induced signals or the surge of DAMPs released from dying parenchymal cells [45]. Pattern-recognition receptors, such as TLR4, NLRP3, and more recently ZBP1, ignite concurrent cell death programs in macrophages, including necroptosis and GSDMD-mediated pyroptosis [46]. Our research indicates that ALOX15 from macrophages facilitates the damage of parenchymal cells via paracrine pyroptosis. Therefore, hematopoietic ALOX15 plays a critical role in multi-organ I/R injury, highlighting the importance of targeting both hematopoietic and parenchymal ALOX15 in conditions related to I/R.

Despite these advances, several limitations warrant consideration. Firstly, our reliance on murine models, particularly in 8-week-old mice, may not fully accurately recapitulate human pathophysiology, especially in aging populations with additional health issues like diabetes or atherosclerosis. Translational validation in large-animal models or human cohorts is imperative. Secondly, although thiolox has shown effectiveness, its pharmacokinetics, optimal dosage, and potential toxicities require further thorough investigation; off-target effects on other lipoxygenases must be ruled out through extensive profiling. Lastly, while genetic knockouts provide initial evidence, they may trigger compensatory changes that pharmacological methods do not. Future research should focus on overcoming these limitations.

To sum up, this work elucidates how ALOX15 regulate pyroptosis through lipid peroxidation and mitochondrial-Ca^2^⁺ signaling, offering a unified paradigm for multi-organ I/R injury. By bridging fundamental mechanisms with therapeutic potential, it paves the way for innovative strategies to maximize reperfusion benefits while minimizing associated risks. Future efforts should validate these findings in clinical settings, which may significantly improve outcomes in cardiovascular and transplant medicine.

## Method

### Mice

WT (Hunan SJA Laboratory Animal Co., Ltd, Changsha, Hunan, China), *Alox15*^*−/−*^ and *Alox15 flox* (GemPharmatech, Nanjing, Jiangsu, China), and *Vav-Cre* and *Casp1/11*^*−/−*^ (Jackson Laboratory, USA) mice, with C57BL/6 J background, age of 8 weeks and weight between 22-25 g, were subjected to various I/R models of an *in-vivo* study. In myocardial I/R model, the mice were anaesthetized using 4.5% isoflurane, and the chest hair was removed. After setting on a 37 ℃ plate supinely, the mice were ventilated with a mixture of oxygen and 1.5% isoflurane. A lateral incision was made in the third intercostal space, and the pericardium was opened to enable access to the heart. The left coronary artery was then ligated for 40 min, followed by reperfusion and closure of the chest. Oxygen ventilation was given until the mice regained consciousness. The hearts were harvested 24 h after the I/R challenge for western blotting (WB) and Even’s blue/TTC staining.

Transient middle cerebral artery occlusion (t-MCAO) was performed as originally described by Longa et al. [47]. Briefly, mice were anesthetied with 1.5% isoflurane in 30% O₂/70% N₂ and maintained normothermic (37.0 ± 0.2 °C) with a servo-controlled heating pad. A silicone-coated 6-0 monofilament (Doccol, USA) was inserted 9-10 mm from the stump of the external carotid artery to occlude the middle cerebral artery. After 60 min, the filament was withdrawn for reperfusion, and the incision was closed. At 24 h post-reperfusion, neurological function was graded in a blinded fashion using the 5-point Longa scale: 0, no deficit; 1, failure to extend contralateral forelimb; 2, circling to the contralateral side when held by tail; 3, falling to the contralateral side; 4, inability to spontaneous movement or loss of consciousness. Subsequently, the brains were then harvested for TTC staining.

In hepatic I/R model, mice were anaesthetized, and a midline incision was made to exposed the liver hilum. Microvascular clips were applied to the left lateral and median lobes for 60 min to induce segmental ischaemia (70% of total liver mass). The abdominal cavity was kept moist with warm saline at 37 °C. Reperfusion was initiated by clip removal, and confirmed visually by color change. Sham-operated animals underwent identical laparotomy without vascular occlusion. Blood samples and liver tissue were harvested 6 h post-reperfusion for downstream analyses.

All mice were housed under standard conditions at room temperature (RT) with a 12 h light-dark cycle, and were free to access to water and standard chow. Animal experiments were preapproved by the Animal Care and Use Committee of the Henan University (DWLL20200109), and were conducted in adherence with the Guidelines of Welfare and Ethics for the Use of Laboratory Animals, China.

### Echocardiography

After anaesthetized, the mice were placed supinely on an electronic plate to continuously monitor the heart rate, respiration, and body temperature. Echocardiography was performed using the Vevo 1100 (VisualSonics Inc.) *in-vivo* imaging system equipped with a 30 MHz probe. M-mode ultrasound was employed to determine the pulsation of the left ventricle, while Doppler ultrasound was used for assessing ventricular function, as indicated by EF and FS.

### Histology analysis and H&E staining

Tissues (liver) were fixed in 4% paraformaldehyde overnight, dehydrated through graded ethanol, cleared in xylene, and then embedded in paraffin. Serial 4 μm sections were deparaffinized and stained with Harris hematoxylin for 3 min, followed by a differentiation in 1% acid ethanol and a counter-staining with eosin for 30 s. After dehydrated, the slides were mounted with DPX and examined under a Nikon Eclipse Ti microscope at a magnification of 10 ×. Infarct areas (necrosis) were quantified in a blinded fashion using ImageJ (NIH).

### Cells

*AC16* cells (immortalized human cardiomyocytes), *HL-1* cells (immortalized mouse cardiomyocytes), *HT-22* (immortalized mouse neurons), *AML-12* (immortalized mouse hepatocytes) and primary mouse peritoneal macrophages were used in the *in-vitro* model of the present study. For silencing *Alox15* or *Hif-1α*, 50 nM of designed siRNAs from commercial kits (Ribobio, Guangzhou, Guangdong, China) were transfected into the cells 48 h before stimulation. For overexpressing *Alox15*, pcDNA3.1 plasmid containing coding region of *Alox15* were transfected into cells (1 μg/mL) using Lipo3000 (Thermo). Cells were subsequently treated with puromycin for 4 weeks before further experiments. In the H/R model, cells were plated and cultured in medium containing 10% fetal bovine serum until reaching 80% confluency. Hypoxia was conducted for 6 h using a gas mixture (O_2_/N_2_/CO_2_, 1:94:5), followed by reoxygenation for 1 h. Cytotoxicity was assessed by determining the percentage of LDH release (Beyotime, Shanghai, China), cell viability was measured using CCK8 kits (Beyotime), and cell death was detected by PI staining (Beyotime) according to the protocols of manufacturers. In additional experiments, cells were harvested for WB and qRT-PCR. Quantification of the oxidative stress marker MDA and ROS were performed using Lipid Peroxidation MDA Assay Kit (Beyotime) and Reactive Oxygen Species Assay Kit (Beyotime), respectively. JC-1 and Fluo-4 AM (Beyotime) were used for determining ΔΨm and cytosolic Ca^2^⁺, respectively.

ASC speckle formation, a hallmark of canonical inflammasome activation, was assessed by immunofluorescence staining. Briefly, cells were fixed with 4% paraformaldehyde at room temperature (RT) for 10 min, followed by permeabilization in 1 × phosphate-buffered saline (PBS) containing 5% BSA and 0.2% Triton X-100 at RT for 10 min. After blocking with 1 × PBS containing 5% BSA for 1 h, cells were incubated with anti-ASC primary antibody (1:100) at 4 °C overnight in a humidified chamber. Subsequently, cells were stained with Alexa Fluor 488-conjugated secondary antibody (1:400) at RT for 1 h in the dark. Finally, nuclei were counterstained with DAPI (1 µg/mL) for 5 min at RT prior to mounting. All steps were separated by three washes with PBS.

### Flow cytometry

Cells were washed twice in PBS containing 2% FBS. Annexin V-FITC (5 μL per 10⁶ cells) and PI (1 μg/mL) were added in Annexin-V binding buffer (10 mM HEPES, 140 mM NaCl, 2.5 mM CaCl₂) for 15 min at RT in the dark. Samples were analyzed within 30 min on a BD FACSCanto II (BD Biosciences). Compensation was performed with single-stained controls. Viable cells (Annexin V⁻/PI⁻) and necrotic (PI⁺) populations were gated using FlowJo v10.8.

### Quantitative real-time polymerase chain reaction

Total RNA was extracted from cells and transcribed into complementary DNA using the Transcriptor First Strand cDNA Synthesis Kit (Invitrogen). The expression levels of the corresponding genes were assessed by qRT-PCR (Vazyme Biotech, Nanjing, Jiangsu, China) with designed primers (see Supplementary Table 1) following a standard protocol. The amplification cycles of GAPDH were used for the normalization of target genes, and the fold changes were calculated as 2^−ΔΔCt^ in comparison to the negative control.

### WB

Lysates from tissues or cells were loaded on SDS-polyacrylamide gel to separate proteins via electrophoresis. The proteins were transferred to PVDF membranes that were then blocked by using 5% fat-free milk. Following an overnight incubation with primary antibodies (see Supplementary Table 2) at 4 ℃, the membranes were incubated with HRP-conjugated secondary antibodies at RT for 2 h. Washes were conducted four times by TBST between each step. Western Bright ECL-Spray was added to the membranes to visualize the blots using a Bio-Rad system.

### Serological analyses and cytokine

The levels of CK and cTnI were determined using commercial kits from Nanjing Jiancheng Bioengineering Institute. Mouse IL-1β (Invitrogen) and IL-18 (Invitrogen) were detected using commercial ELISA kits according to the manufacturers’ instruction.

### Statistical analysis

GraphPad Prism 7.0 software was used for statistical analysis and data display. As dots shown in figures, three independent biological repeats were conducted in *in-vitro* study, where around 5 or 10 mice per group were applied in *in-vivo* study. A Student *t*-test was applied for comparisons between two groups, while One-way or Two-way ANOVA with post-hoc tests was conducted for comparisons among more than two groups. *P*-values less than 0.05 were set as significant difference.

## Supplementary Information

Supplementary Material 1.

Supplementary Material 2.

## Data Availability

All data of the present study are available from the corresponding author on reasonable request.
