# Supplementary material for: 15-lipoxygenase blockade switches off pan-organ ischaemia-reperfusion injury by inhibiting pyroptosis
Source: Mol Biomed. 2025 Oct 10;6:77. doi: 10.1186/s43556-025-00325-z (PMC12511505; doi:10.1186/s43556-025-00325-z)
Supplement: Supplementary file 1 — Supplementary Material 1. [file 43556_2025_325_MOESM1_ESM.docx]

Supplementary Materials for

15-Lipoxygenase Blockade Switches Off Pan-Organ Ischaemia–Reperfusion Injury by Inhibiting Pyroptosis

**Author information**

Jie Li^1,^*, Hailong Zhang^2^, Mengmeng Dai^2^, Yongpan Huang^3^

Author affiliations: 1. Central Laboratory, The Affiliated Changsha Hospital of Xiangya School of Medicine, Central South University, Changsha, 410005, China; 2. Joint National Laboratory for Antibody Drug Engineering, School of Medicine, Henan University, Kaifeng 475004, China; 3. Medical School, Changsha Social Work College, Changsha, 410004, China

*Correspondence: Jie Li, email: li_jie_1983@163.com

ORCID

Jie Li: https://orcid.org/0009-0009-4345-3951

**This word file includes:**

Tables. S1, S2, S3.

Table S1

Table S1 Primers used in qRT-PCR

| Genes | Sequences of primers |
| --- | --- |
| Alox15 | GGCTCCAACAACGAGGTCTAC |
|  | AGGTATTCTGACACATCCACCTT |
| Nlrp3 | ATTACCCGCCCGAGAAAGG |
|  | TCGCAGCAAAGATCCACACAG |
| Asc | CTTGTCAGGGGATGAACTCAAAA |
|  | GCCATACGACTCCAGATAGTAGC |
| Caspase-1 | ACAAGGCACGGGACCTATG |
|  | TCCCAGTCAGTCCTGGAAATG |
| Caspase-11 | ACAAACACCCTGACAAACCAC |
|  | CACTGCGTTCAGCATTGTTAAA |
| Gsdmd | CCATCGGCCTTTGAGAAAGTG |
|  | ACACATGAATAACGGGGTTTCC |
| Gapdh | AGGTCGGTGTGAACGGATTTG |
|  | TGTAGACCATGTAGTTGAGGTCA |

Table S2

Table. S2 Antibodies used in WB and immunofluorescence

| Protein | Manufacturers | Cat. NO. | Dilution |
| --- | --- | --- | --- |
| ALOX15 | Cell Signaling Technologies | 82129S | 1:1000 |
| GSDMD | Abcam | ab209845 | 1:1000 |
| β-ACTIN | Cell Signaling Technologies | 3700S | 1:1000 |
| CASP11 | Abmart | TN23724M | 1:1000 |
| ASC | Cell Signaling Technologies | 67824T | 1:100 |

Table S3

Table. S3 List of 190 chemicals

|  | 1 | 2 | 3 | 4 | 5 | 6 | 7 | 8 | 9 | 10 | 11 | 12 |
| --- | --- | --- | --- | --- | --- | --- | --- | --- | --- | --- | --- | --- |
| 1 |  | Azithromycin | Colchicine | Methyl Vanillate | Levofloxacin hydrate | 3,5-Dimethoxyphenol | Caffeic Acid Phenethyl Ester | Trifolirhizin | Bestatin | 2-Phenylbutyric acid | L-Glutamine | DL-Citrulline |
| 2 | Sulfameter | Ruxolitinib Phosphate | Glabridin | 6-Hydroxyflavone (6-HF) | Dapson | Monomethyl glutarate | Puromycin 2HCl | Tabersonine | Prilocaine | Imidazole-4(5)-acetic Acid Hydrochloride | Penicillamine | Homotaurine |
| 3 | Erythromycin | Oridonin | Cyclosporin A | Cefamandole nafate | Cefuroxime sodium | 3-(3-Hydroxyphenyl)propionic Acid | BTB06584 | Brazilin | Prednisone | Isethionic acid sodium salt | Octenidine Dihydrochloride | Calcium folinate |
| 4 | Fangchinoline | (-)-Parthenolide | Cytisine | Sulfamonomethoxine | Tedizolid Phosphate | Phenylacetylglutamine | Erythromycin Cyclocarbonate | Lycorine hydrochloride | Acetylcysteine | (S)-2-Hydroxy-3-phenylpropanoic acid | D-glutamine | D-Pantethine |
| 5 | Amphotericin B | Rotenone (Barbasco) | Daidzin | Thiolox | Cefmenoxime hydrochloride | Cytidine 5′-triphosphate (disodium salt) | Piperlongumine | Dehydrocorydalin | Ethinyl Estradiol | Glycerol Tri-n-octanoate | D-Cycloserine | L-carnosine |
| 6 | Ursodiol | Silymarin | Dihydroartemisinin (DHA) | 4-Amino-5-imidazolecarboxamide | Hydroquinidine | 2-Methylhexanoic acid | Betulonic acid | Ginkgolic Acid | Monobenzone | Glucosamine | ML-351 | β-Nicotinamide Mononucleotide |
| 7 | Nitrofural | Pefloxacin Mesylate | Sorafenib | Spermidine trihydrochloride | Fusidate Sodium | 5-Hydroxymethyl-2-furancarboxylic acid | Abscisic Acid (Dormin） | 8-Gingerol | Loteprednol etabonate | 2-Aminoethanethiol | Clindamycin Phosphate | Cephradine monohydrate |
| 8 | Tretinoin | Sparfloxacin | Tizoxanide | Tauroursodeoxycholic Acid (TUDCA) | Gluconolactone | DL-Dopa | Isoliquiritigenin | 3'-Hydroxypterostilbene | Betamethasone Dipropionate | Pterostilbene | Kanamycin sulfate | L(-)-Sorbose |
| 9 |  | Nicotinamide (Vitamin B3) | Flavopiridol HCl | Cefepime Dihydrochloride Monohydrate | Vitamin E | 6-(Dimethylamino)purine | Dehydrocostus Lactone | Pinoresinol dimethyl ether | Levonorgestrel | Resveratrol | Chondroitin sulfate | 3-Aminopropionitrile fumarate |
| 10 | Estrone | Vitamin B12 | Actinomycin D (Dactinomycin) | 2-Methoxy-1,4-naphthoquinone | Lycorine | 3-Methylvaleric acid | Guggulsterone E&Z | Echinatin | Guaifenesin | Coniferyl alcohol | Aloperine | 1,4-Diaminobutane dihydrochloride |
| 11 | Flucytosine | Metronidazole | Epirubicin HCl | Thymopentin | Escin | Glycodeoxycholic acid sodium salt | Isoalantolactone | Veratramine | Acetylcholine Chloride | Nervonic acid | Tobramycin | Citicholine |
| 12 | Aminophylline | Tioconazole | Tannic acid | Cefsulodin sodium | D panthenol | cis,cis-Muconic acid | Fumagillin | a-Hederin | Proparacaine HCl | Cardamonin | NAD+ | 5'-Cytidylic acid |
| 13 | Amorolfine HCl | Pregnenolone | Genistin (Genistoside) | Cefonicid sodium | Vitamin K1 | Quinoline-4-carboxylic acid | Docetaxel Trihydrate | Nonivamide | Menadione | Docosahexaenoic Acid | Methacycline HCl | NDGA |
| 14 | TBB | Sulfamethoxazole | 2-Methoxy-1,4-naphthoquin | Umbelliferone | Eugenol | 4-MMPB | Licochalcone A | Sibiricose A5 | Diclazuril | Tabersonine hydrochloride | Oxacillin sodium monohydrate | L-Homoarginine hydrochloride |
| 15 | Sulfanilamide | Sulfisoxazole | gossypol-Acetic acid | Cinnamic acid | Oleic Acid | 3-Furoic acid | Oleuropein | Bergaptol | Tiopronin | Ftaxilide | Neomycin sulfate | Sodium succinate |
| 16 | Hydrocortisone | Nystatin (Fungicidin) | Gramine | Flavanone | Amentoflavone | TBHQ | Xanthohumol | Schisantherin B | Mecarbinate | Eicosapentaenoic Acid | Streptomycin sulfate | Kaempferol-3-O-glucorhamnoside |
