# Supplementary material for: 15-lipoxygenase blockade switches off pan-organ ischaemia-reperfusion injury by inhibiting pyroptosis
Source: Mol Biomed. 2025 Oct 10;6:77. doi: 10.1186/s43556-025-00325-z (PMC12511505; doi:10.1186/s43556-025-00325-z)

Supplementary Materials for

15-Lipoxygenase Blockade Switches Off Pan-Organ Ischaemia–Reperfusion Injury by Inhibiting Pyroptosis

**Author information**

Jie Li^1,^*, Hailong Zhang^2^, Mengmeng Dai^2^, Yongpan Huang^3^

Author affiliations: 1. Central Laboratory, The Affiliated Changsha Hospital of Xiangya School of Medicine, Central South University, Changsha, 410005, China; 2. Joint National Laboratory for Antibody Drug Engineering, School of Medicine, Henan University, Kaifeng 475004, China; 3. Medical School, Changsha Social Work College, Changsha, 410004, China

*Correspondence: Jie Li, email: li_jie_1983@163.com

ORCID

Jie Li: https://orcid.org/0009-0009-4345-3951

**This word file includes:**

The full uncropped Blots image(s).

The full uncropped Blots image(s):

The full uncropped Alox15 images (up) and Actin images (down) in Figure 1e.

**ALOX15 75 kDa**

**
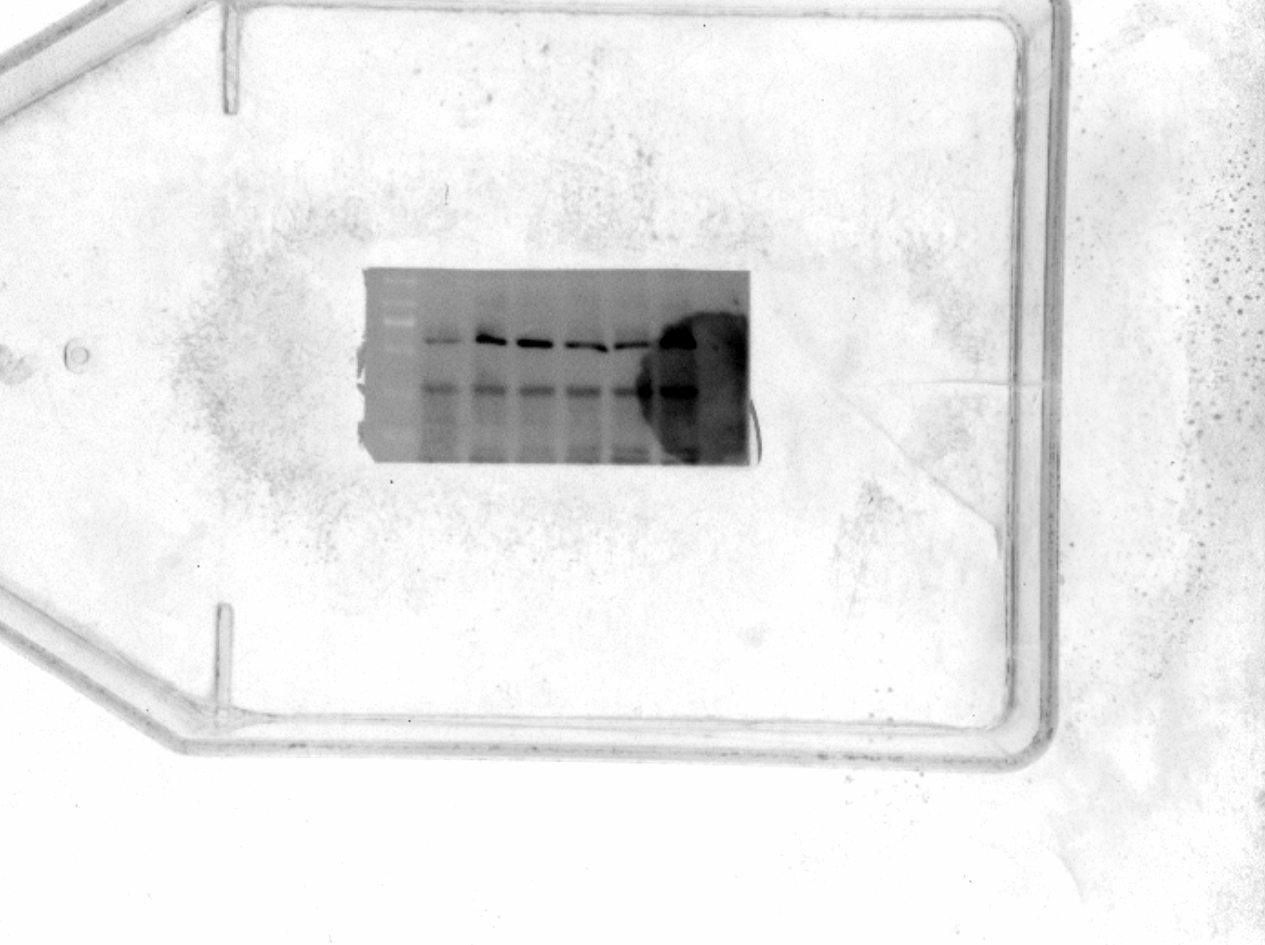
**

**ACTIN 43 kDa**

**
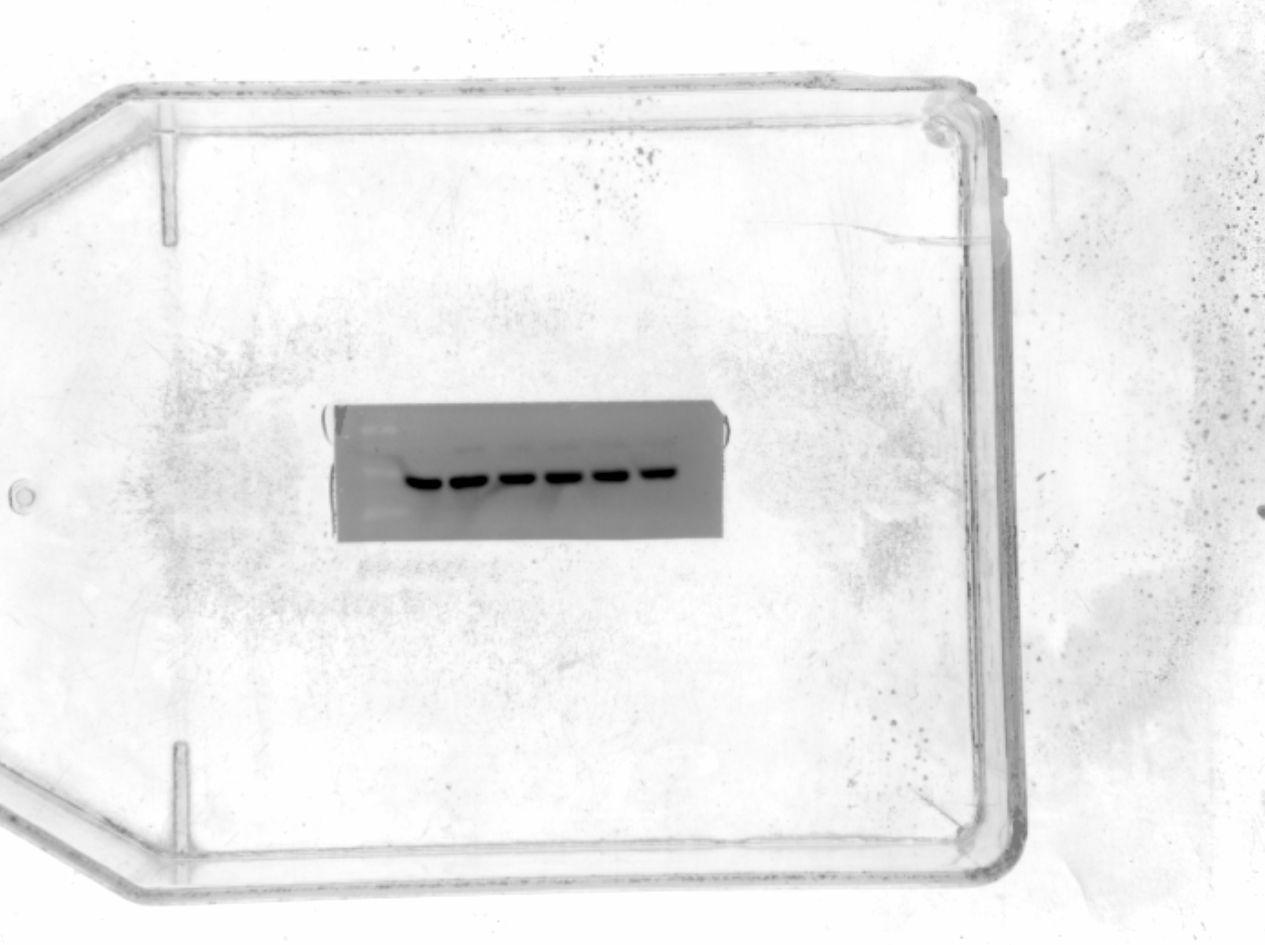
**

The full uncropped GSDMD images (up) and Actin images (down) in Figure 4c.

**GSDMD 53 kDa**

**cl.GSDMD 32kDa**

**
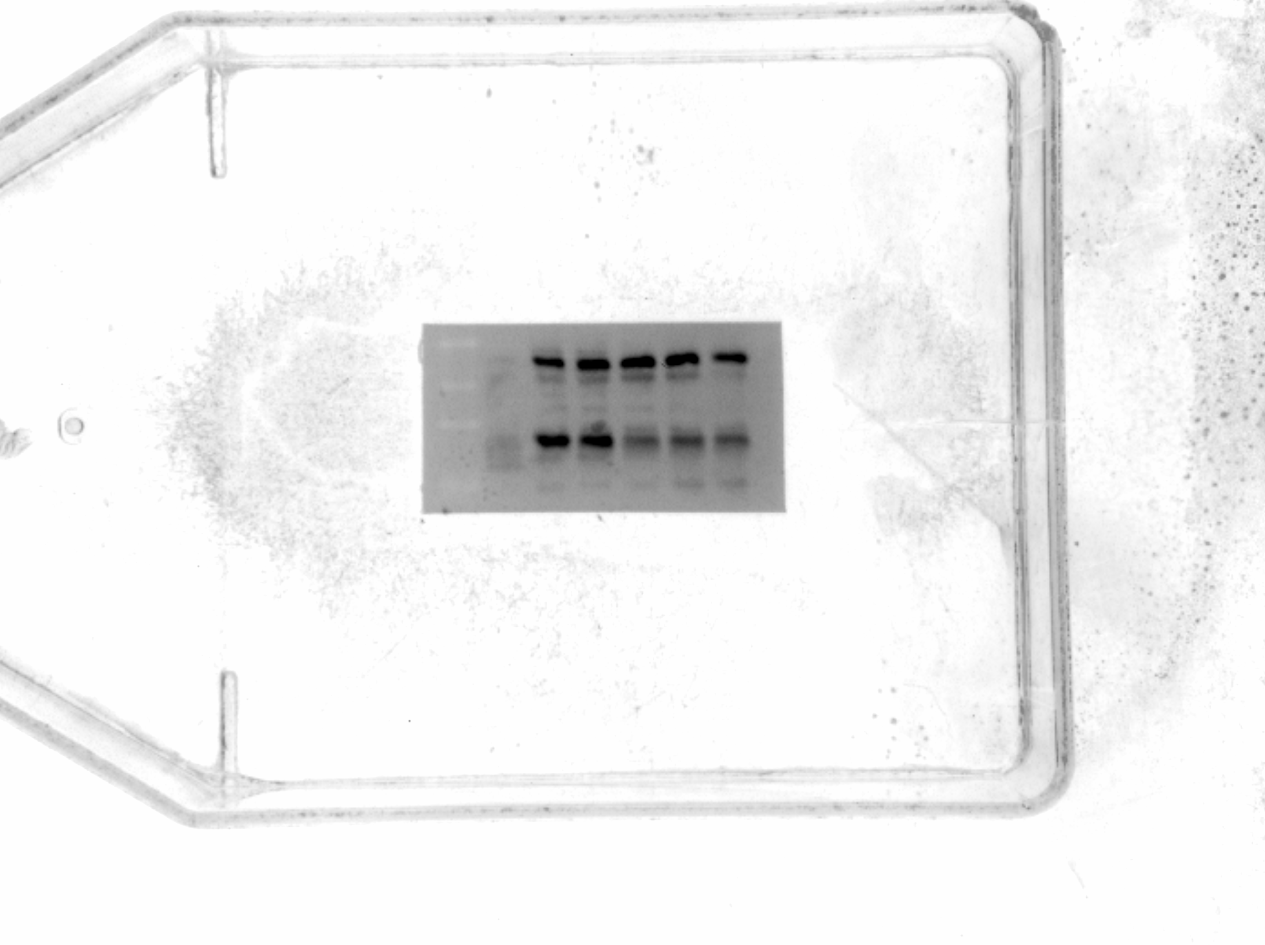
**

**ACTIN 43 kDa**


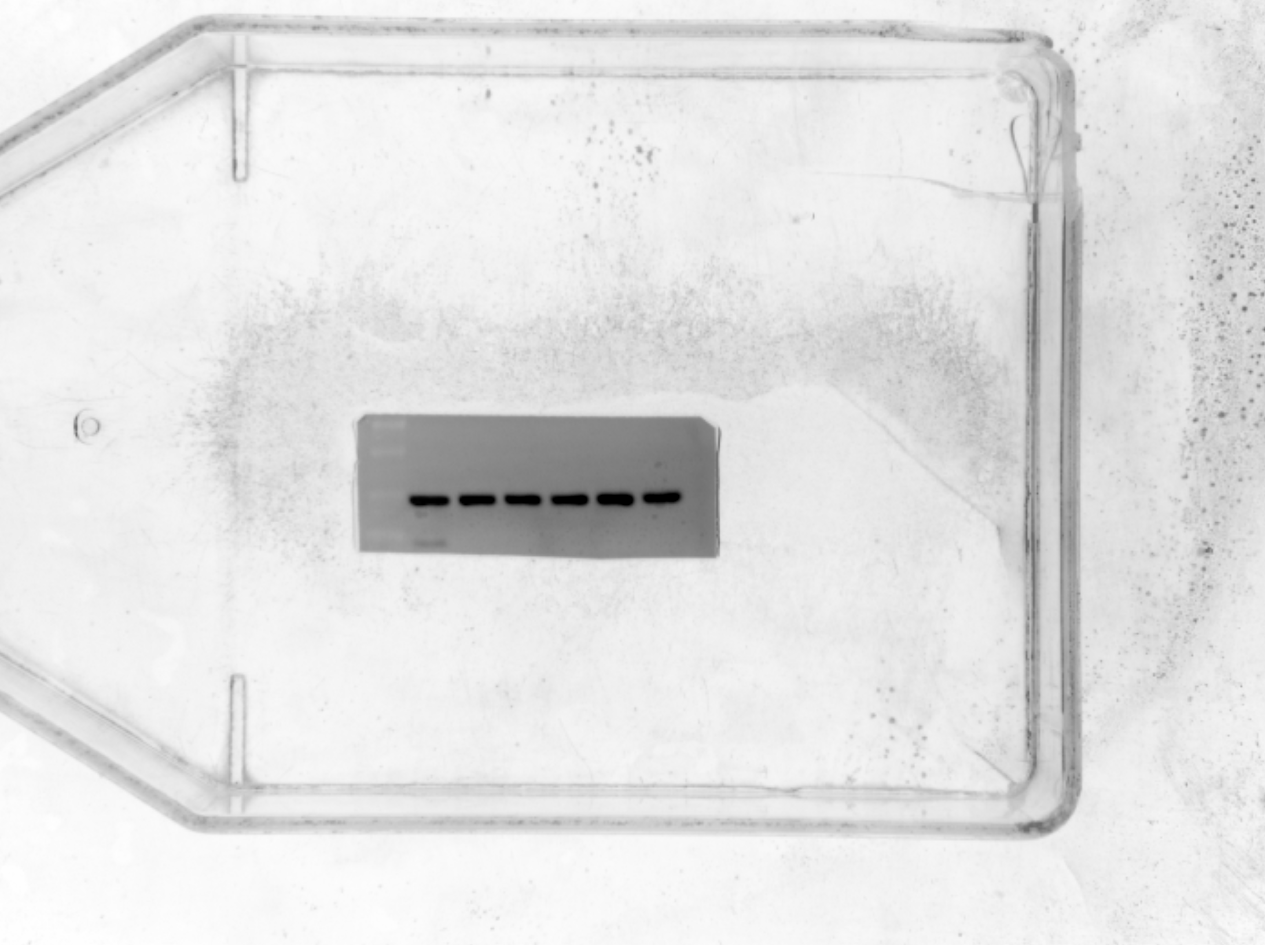


The full uncropped Casp11 images (up) and Actin images (down) in Figure 6e.

**CASP11 IP**


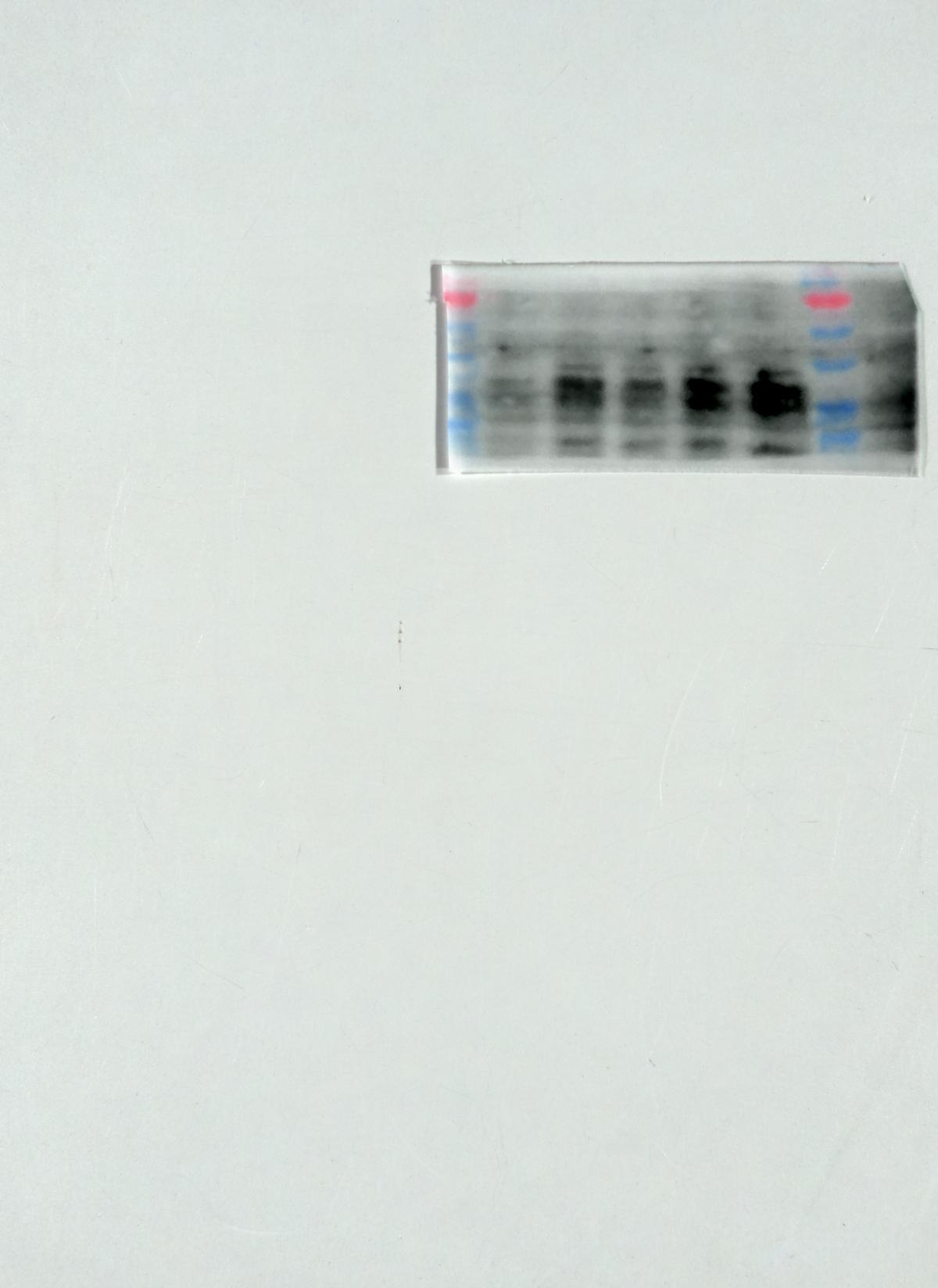


**CASP11 Input**


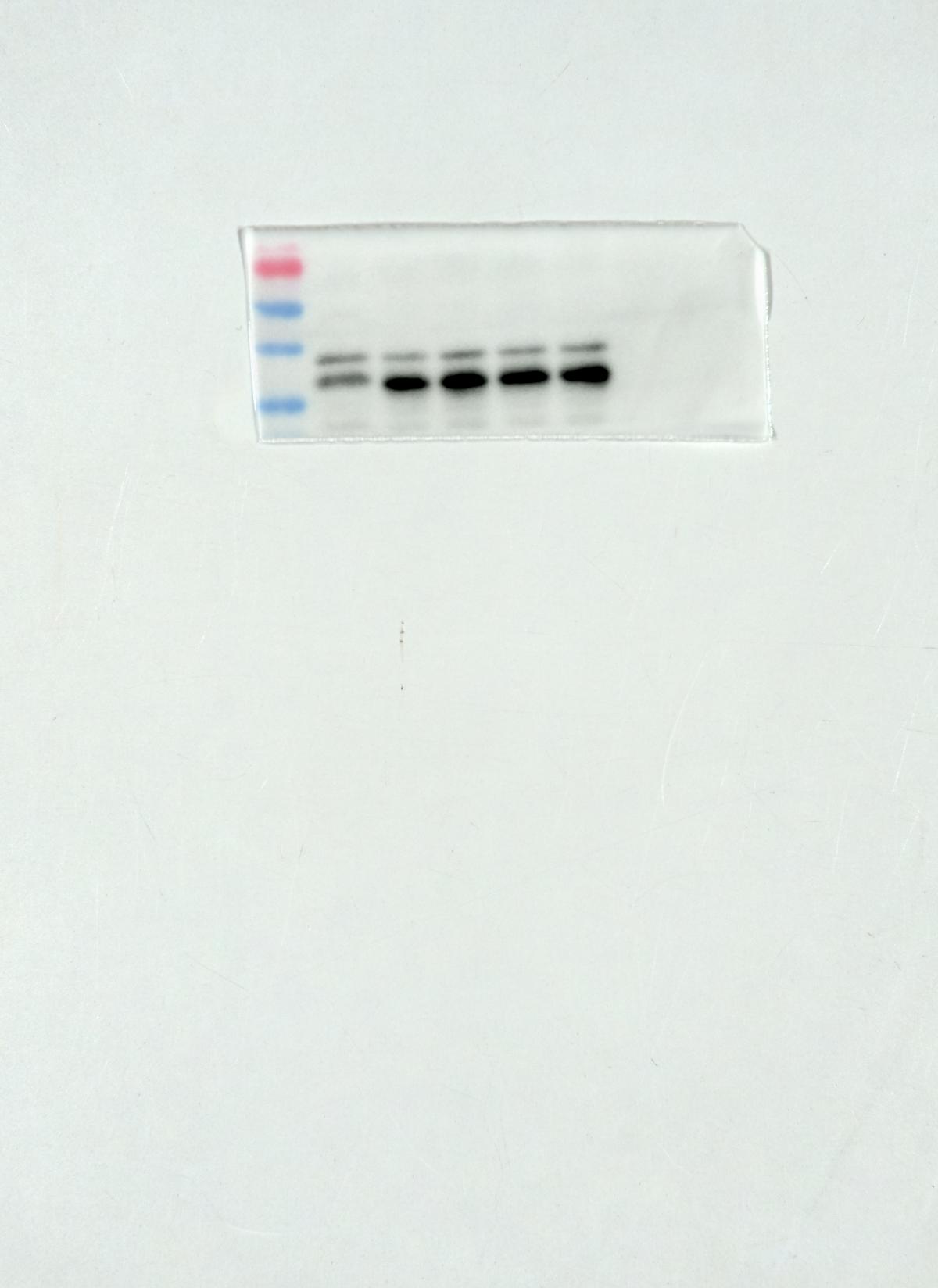


**ACTIN Input 43 kDa**


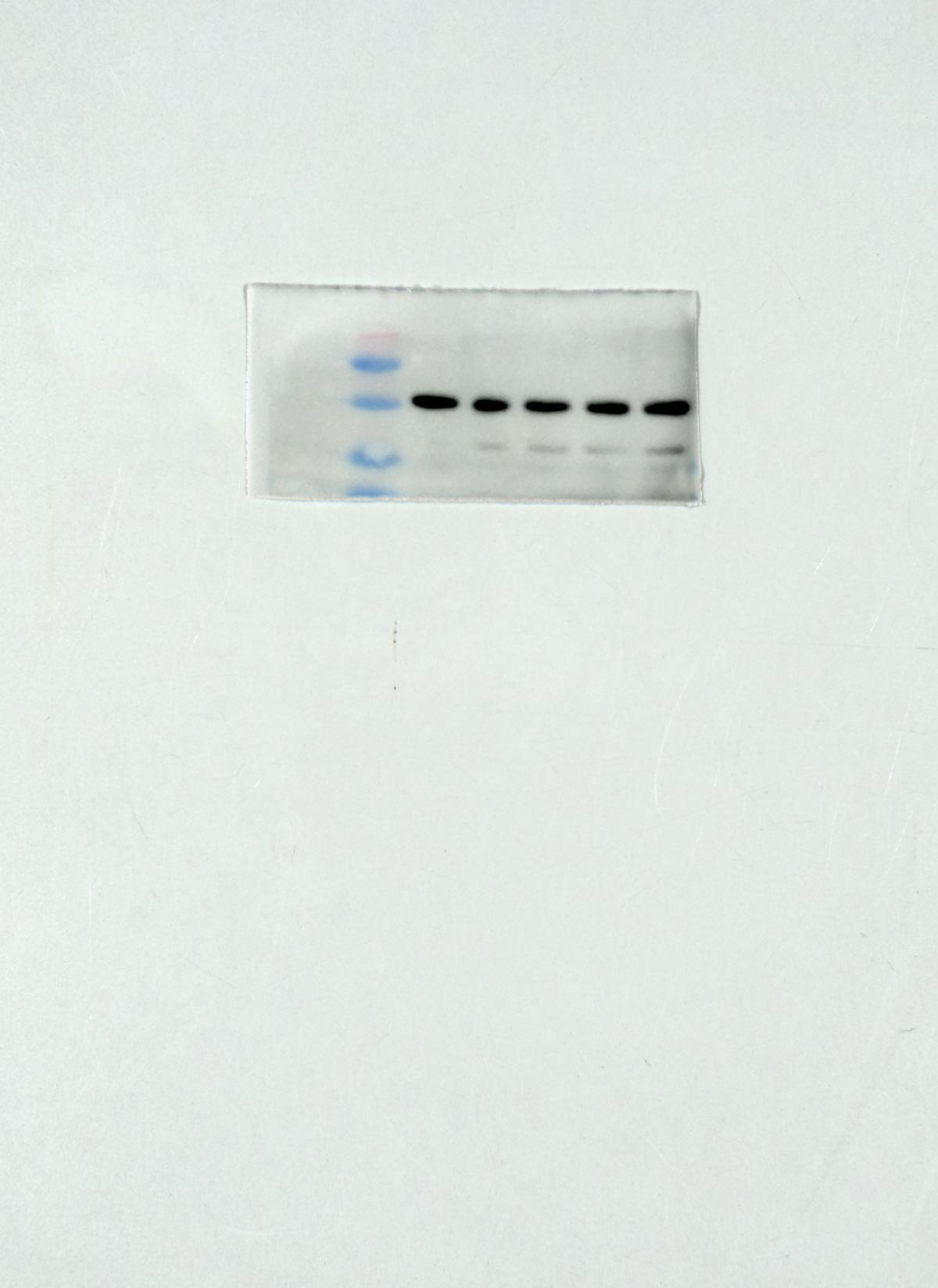

Supplement: Supplementary file 2 — Supplementary Material 2. [file 43556_2025_325_MOESM2_ESM.docx]
